# The rangeomorph *Pectinifrons abyssalis*: Hydrodynamic function at the dawn of animal life

**DOI:** 10.1016/j.isci.2023.105989

**Published:** 2023-01-16

**Authors:** Simon A.F. Darroch, Susana Gutarra, Hale Masaki, Andrei Olaru, Brandt M. Gibson, Frances S. Dunn, Emily G. Mitchell, Rachel A. Racicot, Gregory Burzynski, Imran A. Rahman

**Affiliations:** 1 Department of Earth and Environmental Sciences, Vanderbilt University, Nashville, TN 37240, USA; 2 Evolutionary Studies Institute, Vanderbilt University, Nashville, TN 37235, USA; 3 Senckenberg Research Institute and Natural History Museum Frankfurt, 60325 Frankfurt, Germany; 4 The Natural History Museum, London SW7 5BD, UK; 5 Department of Chemistry and Physical Sciences, University of Toronto Mississauga, Mississauga, ON L5L 1C6, Canada; 6 Oxford University Museum of Natural History, University of Oxford, Oxford OX1 3PW, UK; 7 Department of Zoology, Museum of Zoology, University of Cambridge, Cambridge CB2 3EJ, UK; 8 Department of Biology, Fairfield University, Fairfield, CT 06824, USA

**Keywords:** Zoology, Evolutionary biology, Paleobiology

## Abstract

Rangeomorphs are among the oldest putative eumetazoans known from the fossil record. Establishing how they fed is thus key to understanding the structure and function of the earliest animal ecosystems. Here, we use computational fluid dynamics to test hypothesized feeding modes for the fence-like rangeomorph *Pectinifrons abyssalis*, comparing this to the morphologically similar extant carnivorous sponge *Chondrocladia lyra*. Our results reveal complex patterns of flow around *P. abyssalis* unlike those previously reconstructed for any other Ediacaran taxon. Comparisons with *C*. *lyra* reveal substantial differences between the two organisms, suggesting they converged on a similar fence-like morphology for different functions. We argue that the flow patterns recovered for *P. abyssalis* do not support either a suspension feeding or osmotrophic feeding habit. Instead, our results indicate that rangeomorph fronds may represent organs adapted for gas exchange. If correct, this interpretation could require a dramatic reinterpretation of the oldest macroscopic animals.

## Highlights

• The rangeomorph *Pectinifrons* created unique patterns of fluid flow
• Flow patterns do not support suspension feeding or osmotrophy in *Pectinifrons*
• *Pectinifrons* and the harp sponge converged on similar forms for different functions
• Rangeomorph fronds might be organs adapted for gas exchange

## Graphical abstract

**Figure:**
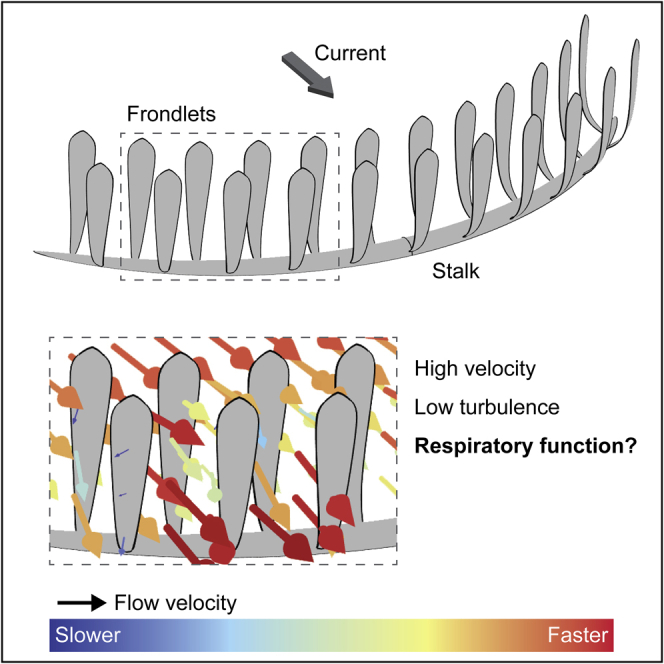

## Introduction

The Rangeomorpha are an enigmatic late Ediacaran (∼575–539 Ma) clade that were integral to the first major radiation of macroscopic eukaryotic life, and which are characterized by a modular mode of construction based on the growth and differentiation of fractal branching frondlets.^1^^,^^2^^,^^3^^,^^4^^,^^5^ Although this unusual fractal mode of construction has led to significant disagreements about how rangeomorphs are related to Metazoa,^1^^,^^6^ recent phylogenetic and developmental approaches^7^^,^^8^ suggest that they may represent stem-eumetazoans. However, while rangeomorphs may be relatively well phylogenetically constrained,^8^ many facets of their paleobiology—including how they fed—are still debated, with a variety of competing hypotheses proposed.^9^^,^^10^ Establishing rangeomorph paleobiology may thus be key to understanding the structure and function of the earliest animal ecosystems, as well as the ecological context underlying the latest Neoproterozoic rise of animals.

**Figure 1:**
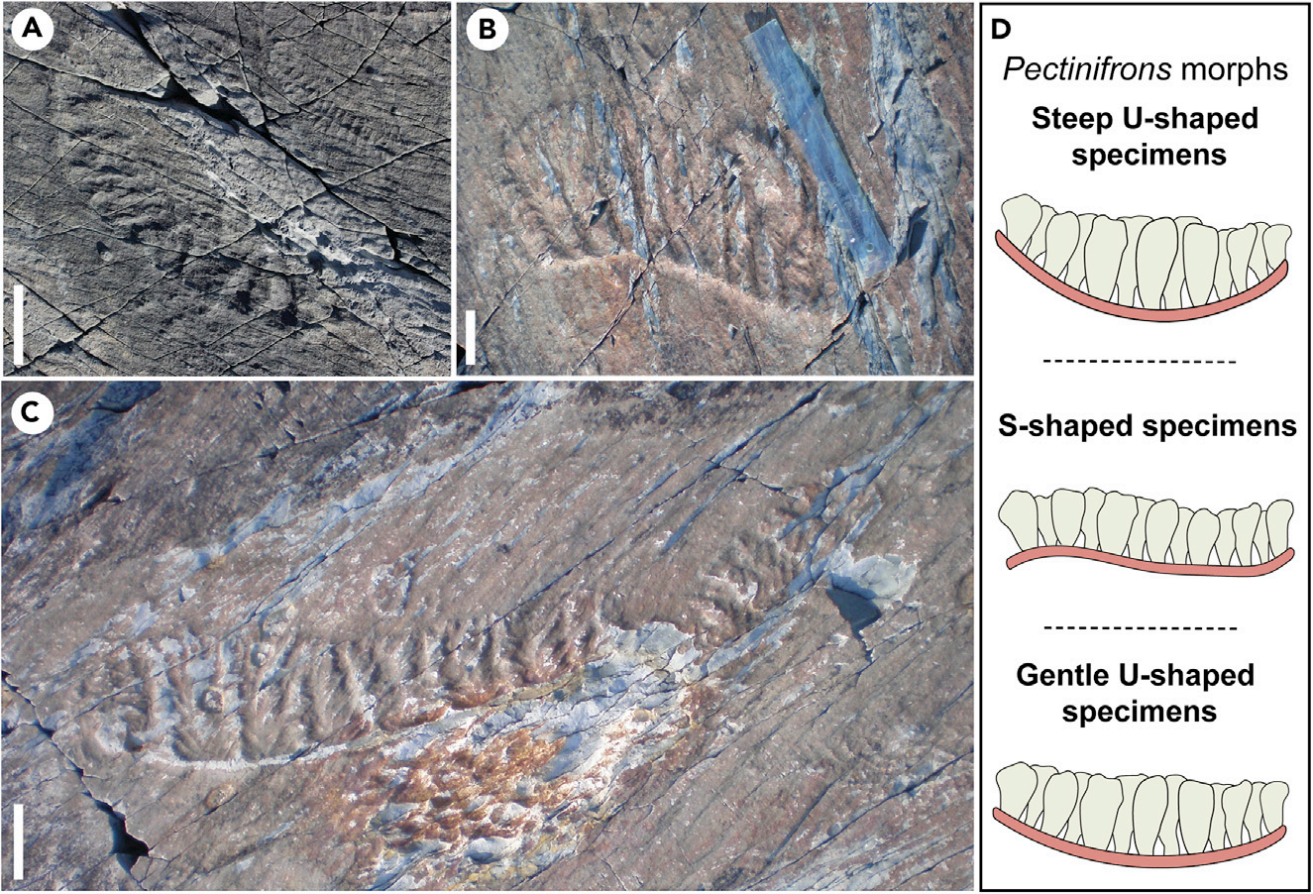
*Pectinifrons abyssalis* from Mistaken Point, Newfoundland, Canada (A–C) Photographs of steep ‘U’-shaped (A), ‘S’-shaped (B), and gentle ‘U’-shaped (C) morphotypes of *Pectinifrons*. Scale bars: 5 cm. (D) Schematic diagrams of *Pectinifrons* morphotypes.

*Pectinifrons abyssalis* is a fence-like rangeomorph (Figure 1) known from the Mistaken Point and Trepassey formations in the Avalon Peninsula, Newfoundland, Canada,^11^ recovered from rocks interpreted as recording deep-marine depositional environments.^12^^,^^13^ Sedimentological studies^12^^,^^13^^,^^14^ have demonstrated that these sections were deposited well below storm wave base (perhaps up to 1.5 km depth^14^), implying that any organisms living in these settings could not have sustained a photoautotrophic metabolism.^15^ *Pectinifrons* has a distinctive architecture that differentiates it from other rangeomorphs, consisting of a basal stalk or stolon that rested on the sediment surface and supported two offset rows of frondlets that extended upward into the water column (Figure 1). Two different morphotypes with ‘U’- or ‘S’-shaped stalks have been recognized^11^ (Figure 1), with ‘U’-shaped morphotypes either gently (Figure 1C) or steeply curved (Figure 1A). Although the fence-like morphology of *Pectinifrons* is unique among rangeomorphs, some extant organisms have adopted a superficially similar body plan and so may offer insights into the ecological and biological advantages of this form. Among these, carnivorous demosponges belonging to the family Cladorhizidae are of equivalent size, and moreover are most common in analogous deep-water settings characterized by oligotrophic conditions and low densities of suspended food particles.^16^^,^^17^ The harp sponge (*Chondrocladia lyra*) is particularly noteworthy in this context, being constructed of between one and six vanes that consist of vertical branches arising from horizontal stolons.^17^^,^^18^

**Figure 2:**
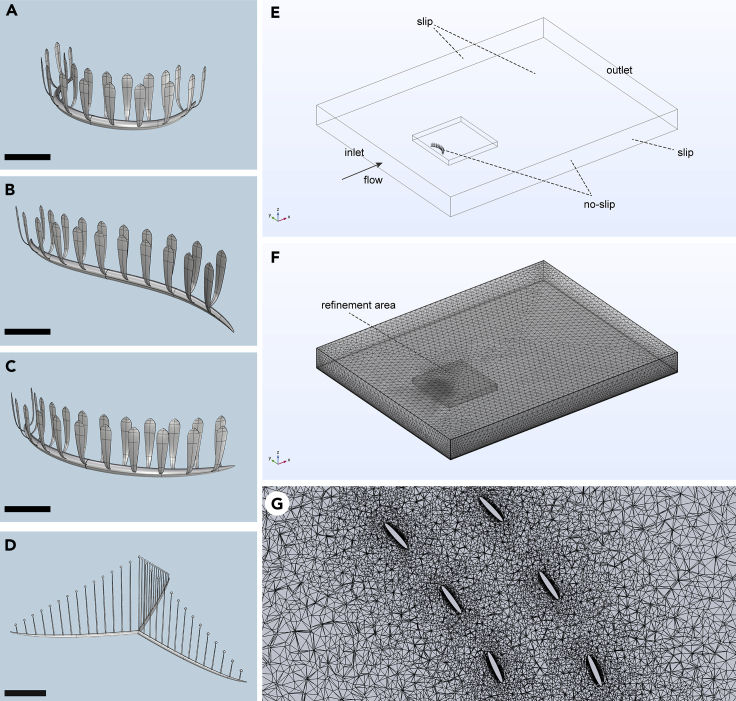
Digital models and computational domain (A–D) Digital models of steep ‘U’-shaped (A), ‘S’-shaped (B), and gentle ‘U’-shaped (C) morphotypes of *Pectinifrons* and three-vaned *C*. *lyra* (D). (E and F) Computational domain used in CFD simulations. (G) Horizontal cross-section (*z* = 2 cm) through mesh used in CFD simulations for refinement area shown in (F). Scale bars: 10 cm. See also Figure S1.

In this study, we use computational fluid dynamics (CFD) to reconstruct patterns of fluid flow around digital models of the Ediacaran rangeomorph *Pectinifrons* and the extant sponge *C. lyra* (Figure 2). We use the results to test hypothesized feeding modes in *Pectinifrons*, shedding new light on the ecological role of rangeomorphs in late Neoproterozoic deep-water benthic ecosystems. Moreover, by comparing reconstructed flow patterns with those obtained for *C. lyra*, we test the extent to which organisms separated by over half a billion years have converged on similar fence-like morphologies as adaptations for a common function.

### Computational fluid dynamics and inference of feeding modes

CFD is a numerical approach for simulating fluid flow, which has applications for studying the function and ecology of a wide range of extinct organisms.^19^^,^^20^^,^^21^ In recent years, CFD has increasingly been used to analyze the paleobiology of Ediacaran fossils,^21^^,^^22^^,^^23^^,^^24^^,^^25^^,^^26^^,^^27^ leveraging the observation that organisms living in modern marine environments have evolved morphologies and behaviors that allow them to thrive in settings characterized by moving fluids.^28^^,^^29^ This previous work, in combination with a wealth of experimental studies on extant organisms,^29^ has allowed us to establish a robust logic structure for testing hypotheses for the feeding modes of enigmatic fossil taxa.

### Feeding in *Pectinifrons*

As with all rangeomorphs, the feeding mode of *Pectinifrons* is the subject of ongoing debate; there is no evidence that it was mobile, and hence osmotrophy,^9^ suspension feeding,^10^ and chemoautotrophy^30^ have been suggested as likely feeding modes. Both osmotrophy and suspension feeding carry different predictions with respect to the relationship between organismal morphology and fluid flow, which are detailed later in the discussion. In contrast, chemoautotrophy is centered on a hypothesized relationship between Ediacaran organisms and near-ubiquitous seafloor microbial mats,^30^^,^^31^ and hence does not generate any hypotheses that are amenable to testing using CFD. We do, however, consider this model in greater detail in the Discussion section. A key observation is that *Pectinifrons* appears not to have—in contrast to other taxa^32^—adopted a preferred orientation on the Mistaken Point Ecological Reserve bedding planes^12^ (although see Ichaso et al.^13^ for a counter example in the Spaniard’s Bay area), indicating that it must have been capable of feeding regardless of orientation to current.

In osmotrophic feeding, organic compounds are digested extracellularly (often via excretion of hydrolytic enzymes) and then absorbed across cell membranes.^9^^,^^10^^,^^33^ This mode of feeding requires a high surface area to volume ratio (SA/V) of tissues exposed to nutrient pools,^3^ and hence the majority of extant organisms that feed osmotrophically are microscopic (e.g., heterotrophic prokaryotes). Nevertheless, a variety of modern macroscopic organisms, including molluscs, sponges, corals and echinoderms, are thought to facultatively employ osmotrophy—in particular while in larval stages—as a supplemental food source.^34^ The idea that rangeomorphs fed via osmotrophy was brought to prominence by Laflamme et al.^3^ based on the observation that the SA/V of rangeomorphs were comparable to those of extant osmotrophic bacteria (although see Butterfield^10^), coupled with the inference that the Ediacaran deep ocean had an unusually large pool of dissolved organic carbon (DOC) that might have served as a potential food source.^33^^,^^35^^,^^36^ Although there are no extant macroscopic free-living animals that feed primarily through osmotrophy and thus could serve as analogs, organisms that acquired nutrients in this manner would be expected to adopt a morphology and/or position in the water column that distributed flow evenly over their feeding surfaces (see, for example, the distribution of water over *Charniodiscus* fronds^37^), thus maximizing the potential for nutrient entrainment and subsequent uptake.^21^ In addition, osmotrophy often involves the secretion of enzymes for the external digestion of macromolecules,^38^ a function which would be limited at high Reynolds numbers (due to the small Péclet numbers associated with nutrient delivery, where particle advective timescales are much greater than diffusive and uptake timescales; see discussion in Butterfield^10^). Based on this, we predict that the morphology of osmotrophic organisms would preferentially encourage low velocity, low turbulence flow over their fractally patterned tissues, as this would maximize entrainment and lead to enhanced opportunities for dissolved nutrients to be absorbed across membranes.

Suspension feeding entails capturing particles suspended in water using feeding structures, with those particles then ingested. In contrast to osmotrophy, it is a common mode of feeding among a wide range of animals in present-day aquatic environments, and so there are a much greater number of modern analogs to draw upon. The idea that rangeomorphs originally functioned as macroscopic, microphagous suspension feeders goes back several decades,^39^ but has recently been resurrected by Butterfield,^10^ who reconstructed rangeomorphs as possessing a hydrostatic skeleton and chambered construction that helped them feed in this manner. In this model, rangeomorph fronds were perforated with numerous small openings; cilia surrounding these openings helped to move seawater into enclosed cavities, where a diverse microbiome would have assisted with extracellular digestion. Although CFD does not allow us to test many specific tenets of this model, the observation that the majority of extant, sessile suspension feeders have evolved morphologies, behavioral adaptations, and/or attitudes relative to the sediment-water interface that aid in feeding does provide a logic structure for interpreting macro-scale flow patterns. LaBarbera^40^ categorized strategies for suspension feeding into six broad types: (1) ‘scan and trap’, (2) sieving, (3) direct interception, (4) inertial impaction, (5) gravitational deposition, and (6) diffusive deposition. Although there are a number of variations on each theme, these strategies are generally characterized by particular morphological and/or fluid dynamics characteristics that help maximize the efficiency of feeding via the chosen mode. For example, sieving and direct interception frequently involve the capture of particles in a filtering array, and so benefit from having large filters extended into the water column through which large volumes of fluid can pass (see, for example, barnacles,^40^ ophiuroids,^41^ crinoids,^42^ and blackfly larvae^43^). Inertial impaction and gravitational deposition rely more on getting particles to cross flow streamlines, and from there impact the collecting organ; in fluid dynamics terms, these strategies thus benefit from creating localized areas of low velocity and/or low turbulence flow adjacent to the collector (see, for example, bivalves^44^ and corals^45^^,^^46^). Regardless of the ultimate strategy for particle collection, however, many suspension feeders are thought to have benefitted from recirculated flow in the wake of the organism,^20^^,^^47^ which could have directed suspended food particles toward specialized collectors. Lastly, suspension feeders that form gregarious populations frequently create, and take advantage of, areas of downstream turbulence that enhance nutrient delivery (and thus availability) to the entire population (see, for example, *Semibalanus* mussels^48^). Detecting areas of enhanced turbulence and vertical mixing over multiple individuals have thus been used as evidence for inferring gregarious suspension feeding in enigmatic fossil taxa.^24^^,^^25^^,^^26^

The observations and logic structure outlined above allow us to propose specific testable predictions following interpretations of *Pectinifrons* as an osmotroph or a suspension feeder. If *Pectinifrons* was an osmotroph, we predict that its morphology would distribute low velocity, low turbulence flow over the entire surface of the organism in order to maximize the potential for nutrient uptake. In contrast, if *Pectinifrons* was a passive suspension feeder we predict that either: a) parts of the anatomy that potentially housed a collecting apparatus would extend up into the water column where they would intercept large volumes of moving fluid; or, b) its external morphology would redirect flow toward feeding structures and/or generate localized areas of low-velocity and low-turbulence flow that would cause food particles to fall out of suspension. As an organism found in populations on bedding planes,^11^ we can also test the prediction that the morphology of *Pectinifrons* created downstream areas of increased turbulence and vertical mixing that enhanced nutrient delivery to nearby individuals. Lastly, given the observation that *Pectinifrons* had no preferred orientation on most bedding planes,^11^ we test the prediction that patterns of fluid flow thought to aid in feeding were consistent regardless of orientation to current.

### Feeding in *C. lyra*

Although relatively little is known about the biology of deep-sea sponges belonging to the family Cladorhizidae, the reduced aquiferous systems seen in many taxa provide evidence of significant divergence from typical sponge filter feeding strategies.^17^ Moreover, the presence of enclosed crustacean and larval carcasses on branches and stolons provides direct evidence of carnivory.^16^^,^^18^ During feeding, prey items become entangled in the filaments between branches, and are subsequently gradually enveloped and digested by sponge cells migrating to the area of contact.^17^^,^^49^ Lee et al.^18^ suggested that the body form of *C. lyra* (i.e., with parallel and upright branches joined to a horizontal stolon situated near the sediment-water interface) likely evolved to maximize potential contact with mesoplankton, while the variation in vane number and orientation may reflect variability in local hydrodynamics (although this has yet to be explicitly tested). In terms of predictions for CFD, as a passive predator the morphology of *C. lyra* can be hypothesized to have functioned much like a macroscopic filtering array, employing a combination of sieving and direct interception strategies for prey capture (*sensu* LaBarbera^40^). In this light, feeding would likely be optimized in relatively low-turbulence flows that would transport suspended particles through and between branches where they could be caught.

## Results

### Flow velocity and direction

CFD results for *Pectinfrons* are shown in Figures 3, 4, 5, 6, and S3–S13, while those for *C. lyra* are presented in Figures 7, 8, and S14–S20. Although the reconstructed patterns of fluid flow differed between models and orientations, some fundamental aspects were common to all simulations. Upstream of the models, there was a low velocity, viscous sublayer developed near the lower boundary of the domain, where velocity increased rapidly above the no-slip surface (i.e., in agreement with the Law of the Wall^50^). Flow was disrupted proximal to the upstream face of the models, initiating flow separation at the model, which was characterized by a region of low velocity, recirculating flow immediately downstream of the model (i.e., the wake) (Figures 3, 4, 7, 8, S3–S6, and S14–S17). The size and shape of the wake were controlled by the model geometry and orientation. Flow reattachment occurred further downstream of the model.

**Figure 3:**
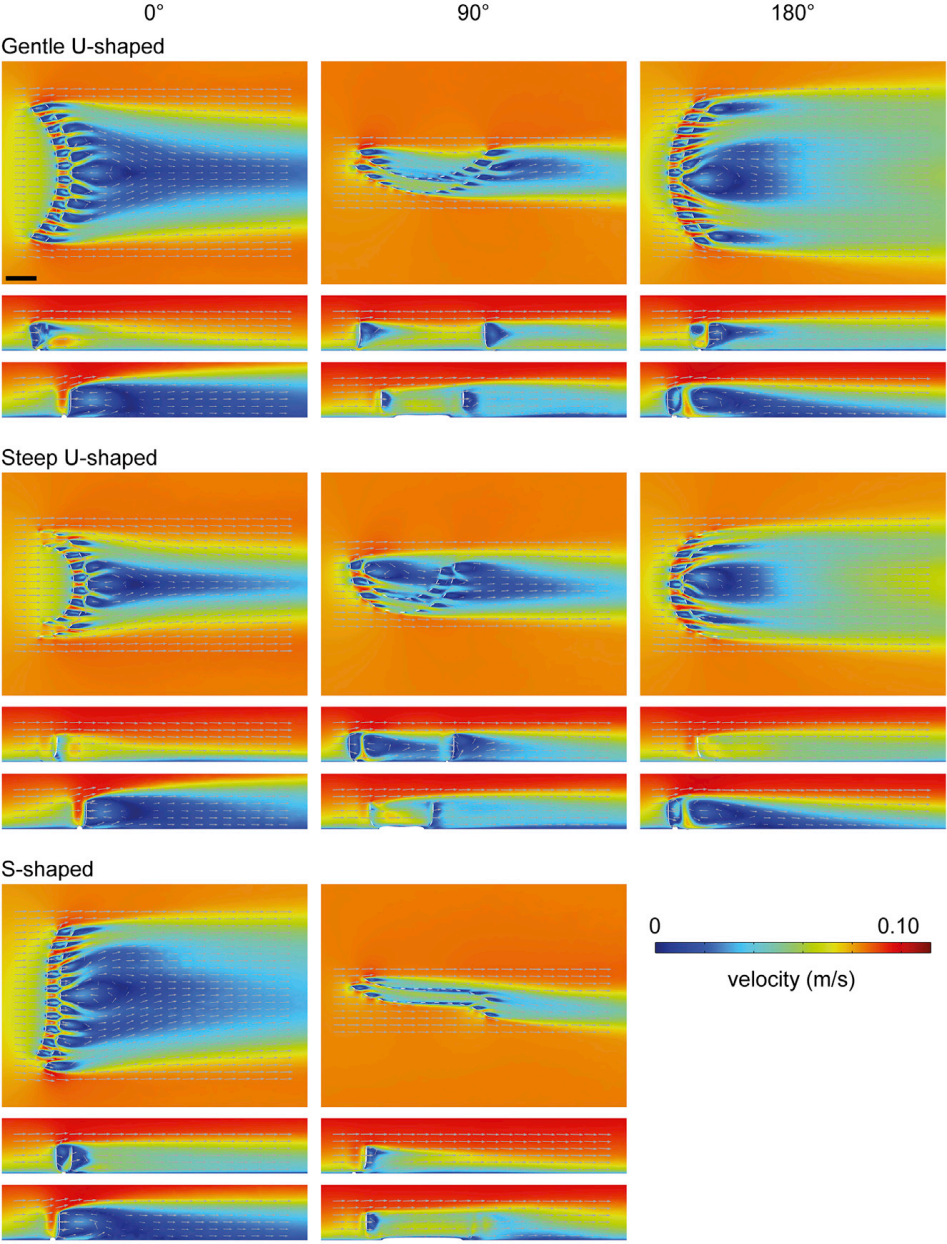
Two-dimensional plots (horizontal and vertical cross-sections) of velocity magnitude (*U*) with flow vectors (size of gray arrows proportional to natural logarithm of velocity magnitude) at an inlet velocity of 0.1 m/s for three *Pectinifrons* models Different *Pectinifrons* morphotypes (i.e., ‘U’- and ‘S’-shaped) are arranged in rows, while models in different orientations to current (i.e., 0°, 90°, and 180°) are arranged in columns. Within each panel, the uppermost plot shows a horizontal cross-section (*z* = 2 cm), while the bottom two panels show vertical cross-sections (gentle ‘U’-shaped model at 0° and 180°, upper plots *y* = 23 cm and lower plots *y* = 0 cm; gentle ‘U’-shaped model at 90°, upper plot *y* = 7 cm and lower plot *y* = 0 cm; steep ‘U’-shaped model at 0° and 180°, upper plots *y* = 18 cm and lower plots *y* = 0 cm; steep ‘U’-shaped model at 90°, upper plot *y* = 7 cm and lower plot *y* = 0 cm; ‘S’-shaped model at 0°, upper plot *y* = 22 and lower plot *y* = 0 cm; ‘S’-shaped model at 90°, upper plot *y* = 5 cm and lower plot *y* = 0 cm). Direction of ambient flow in each panel is from left to right. Scale bar: 10 cm. See also Figures S3 and S4.

**Figure 4:**
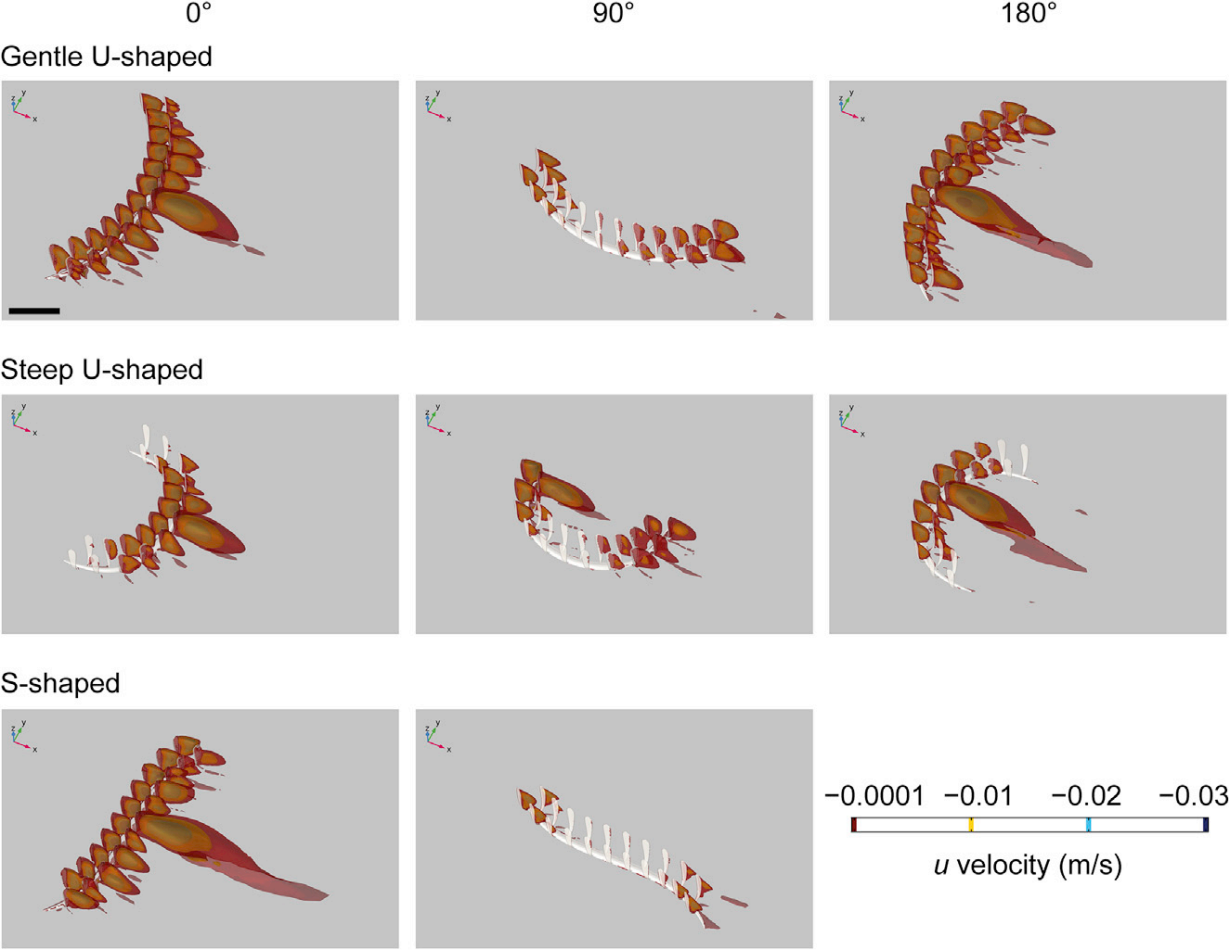
Three-dimensional isosurface plots of negative values of velocity component *u* (streamwise velocity) at an inlet velocity of 0.1 m/s for three *Pectinifrons* models Different *Pectinifrons* morphotypes (i.e., ‘U’- and ‘S’-shaped) are arranged in rows, while models in different orientations to current (i.e., 0°, 90°, and 180°) are arranged in columns. Results for our ‘S’-shaped model at 0° and 180° are identical, and so the latter is not shown. Direction of ambient flow in each panel is from top left to bottom right. Scale bar: 10 cm. See also Figures S5 and S6.

**Figure 5:**
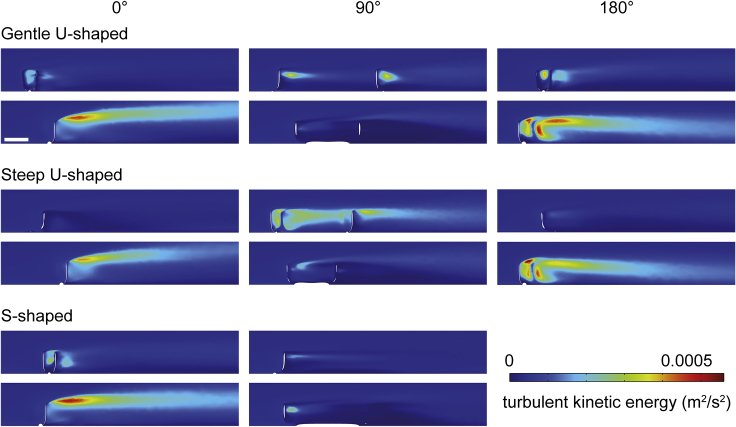
Two-dimensional plots (vertical cross-sections) of turbulent kinetic energy magnitude (*k*) at an inlet velocity of 0.1 m/s for three *Pectinifrons* models Different *Pectinifrons* morphotypes (i.e., ‘U’- and ‘S’-shaped) are arranged in rows, while models in different orientations to current (i.e., 0°, 90°, and 180°) are arranged in columns. Within each panel, the uppermost plot shows a vertical cross-section toward the margin of the model (gentle ‘U’-shaped model at 0° and 180°, *y* = 23 cm; gentle ‘U’-shaped model at 90°, *y* = 7 cm; steep ‘U’-shaped model at 0° and 180°, *y* = 18 cm; steep ‘U’-shaped model at 90°, *y* = 7 cm; ‘S’-shaped model at 0°, *y* = 22; ‘S’-shaped at 90°, *y* = 5 cm), while the lower plot shows a vertical cross-section at the approximate center of the model (*y* = 0 cm). Direction of ambient flow in each panel is from left to right. Scale bar: 10 cm. See also Figures S7 and S8.

**Figure 6:**
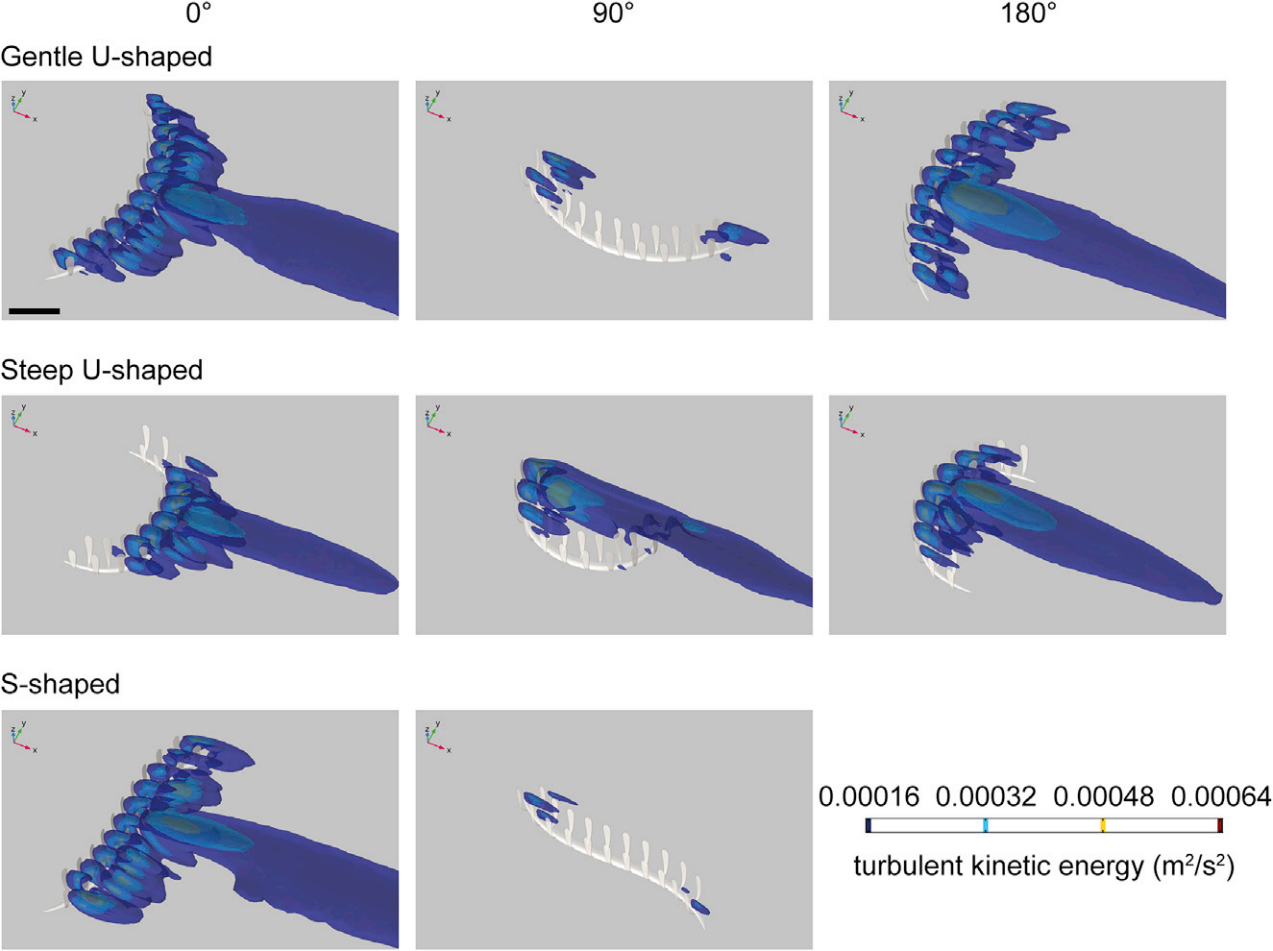
Three-dimensional isosurface plots of turbulent kinetic energy magnitude (*k*) at an inlet velocity of 0.1 m/s for three *Pectinifrons* models Different *Pectinifrons* morphotypes (i.e., ‘U’- and ‘S’-shaped) are arranged in rows, while models in different orientations to current (i.e., 0°, 90°, and 180°) are arranged in columns. Results for our ‘S’-shaped model at 0° and 180° are identical, and so the latter is not shown. Direction of ambient flow in each panel is from top left to bottom right. Scale bar: 10 cm. See also Figures S9 and S10.

**Figure 7:**
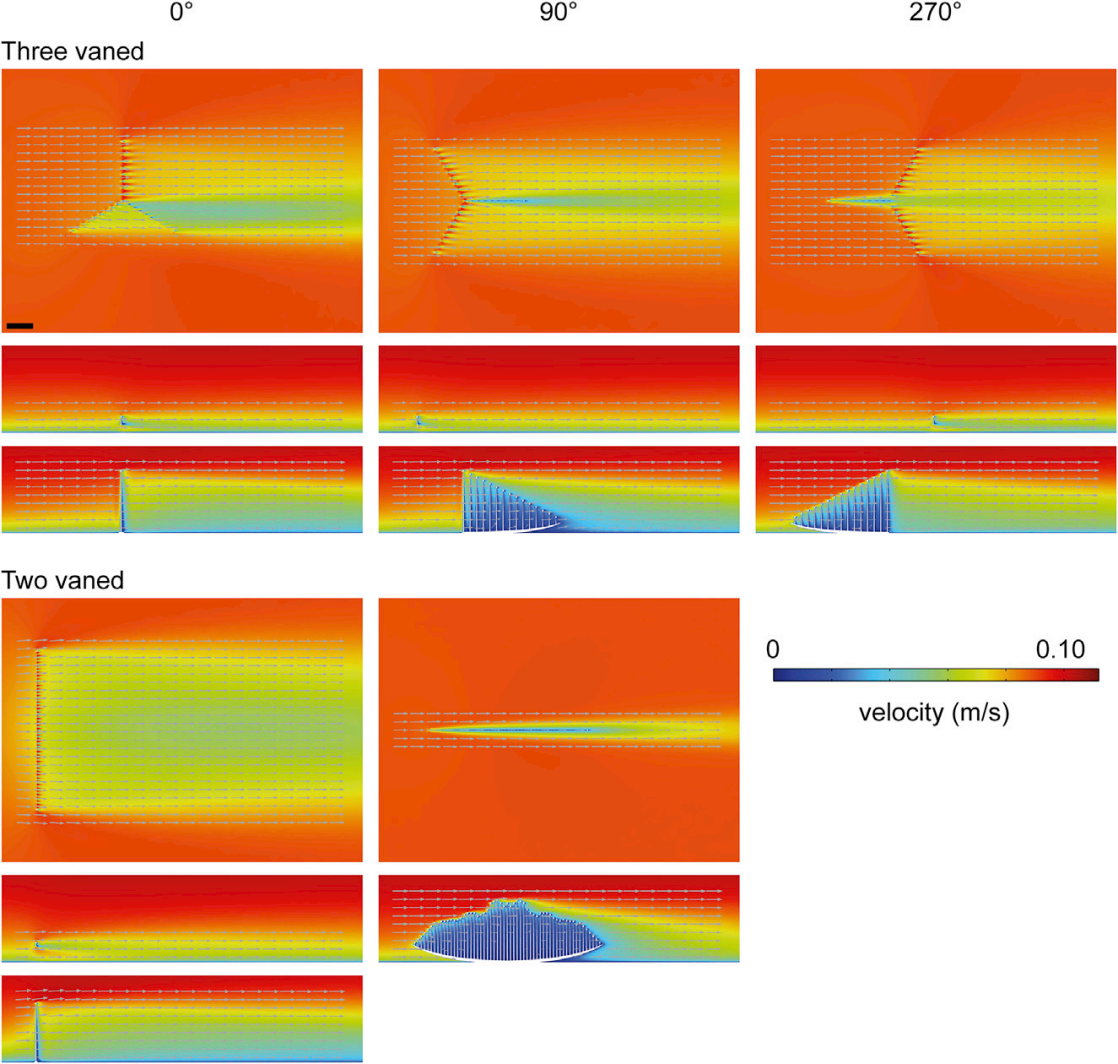
Two-dimensional plots (horizontal and vertical cross-sections) of velocity magnitude (*U*) with flow vectors (size of gray arrows proportional to natural logarithm of velocity magnitude) at an inlet velocity of 0.1 m/s for two *C. lyra* models *C. lyra* morphotypes (i.e., three- and two-vanes) are arranged in rows, while models in different orientations to current (i.e., 0°, 90° and 270°) are arranged in columns. Within each panel, the uppermost plot shows a horizontal cross-section (*z* = 5 cm), while the bottom two panels show vertical cross-sections (three vaned model at 0°, upper plot *y* = 28 cm and lower plot *y* = 0 cm; three vaned model at 90° and 270°, upper plots *y* = 24 cm and lower plots *y* = 0 cm; two vaned model at 0°, upper plot *y* = 28 cm and lower plot *y* = 0 cm; two vaned model at 90°, *y* = 0 cm). Results for our two-vaned model at 0° and 180° are identical, and so the latter is not shown. Direction of ambient flow in each panel is from left to right. Scale bar: 10 cm. See also Figures S14 and S15.

**Figure 8:**
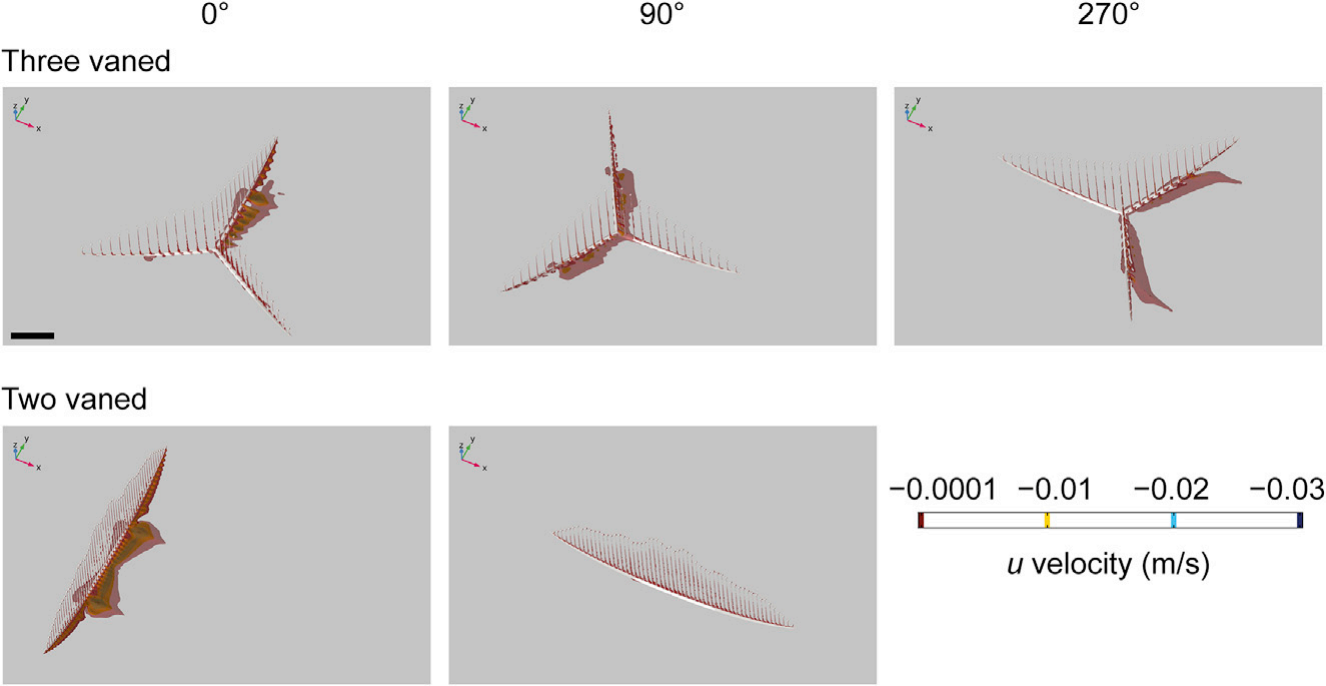
Three-dimensional isosurface plots of negative values of velocity component *u* (streamwise velocity) at an inlet velocity of 0.1 m/s for two *C. lyra models* *C. lyra* morphotypes (i.e., three- and two-vanes) are arranged in rows, while models in different orientations to current (i.e., 0°, 90°, and 180°) are arranged in columns. Results for our two-vaned model at 0° and 180° are identical, and so the latter is not shown. Direction of ambient flow in each panel is from top left to bottom right. Scale bar: 10 cm. See also Figures S16 and S17.

In all the *Pectinifrons* models oriented at 0° or 180° to the inlet, flow accelerated through the gaps between the frondlets (Figures 3, S3, and S4). In the models oriented at 90° to the inlet, flow accelerated between the frondlets at the upstream end of the model, but was then slowed as it passed across the model surfaces, forming a low-velocity zone between the two rows of frondlets (Figures 3, S3, and S4). In the models of *C*. *lyra*, flow acceleration occurred between the vertical branches when the vanes were oriented perpendicular or at an oblique angle to the inlet, but this was not observed for any vanes parallel to the flow direction (Figures 7 and 8). At orientations of 0° or 180° to the inlet, the wakes developed downstream of the ‘U’-shaped *Pectinifrons* and two and three-vaned *C*. *lyra* models were broadly symmetrical (Figures 3 and 8), whereas a more asymmetric wake with an elongate low-velocity zone deflected to the left of the model was observed for the ‘S’-shaped model (Figures 3, S3, and S4). At an orientation of 90° to the inlet, the wake was asymmetrical for all the models (Figures 3, S3, and S4). In the ‘U’-shaped *Pectinifrons* models oriented at 0° to the inlet, there was a single wake developed behind models, which narrowed somewhat as the inlet velocity increased (Figures 3, S3, and S4). At an orientation of 180°, however, this wake split, with one relatively broad lower-velocity zone developed behind the center of the model, and two narrower zones developed toward the lateral margins (Figures 3, S3, and S4). Strong recirculation within the wake was visible in the ‘U’ and ‘S’-shaped *Pectinifrons* models when oriented at 0° or 180° to the inlet (Figures 3, 4, and S3–S6), with flow redirected back toward the frondlets in the center of the models; this effect was stronger at higher simulated inlet velocities (Figures 3, 4, and S3–S6). In the ‘S’-shaped model, recirculation was slightly offset toward the left margin of the model, where it was bent in the streamwise direction (Figures 3, 4, and S3–S6). There was little, if any, recirculation evident toward the lateral margins of the models. Additionally, when the *Pectinifrons* models were oriented at 90° to the inlet, there was comparatively little recirculation, with the exception of the steep ‘U’-shaped model, where recirculation was seen within the middle of the model (i.e., in the space between the curved margins) (Figures 3, 4, and S3–S6). Regardless of orientation, no recirculation was observed for either of the *C*. *lyra* models at all simulated inlet velocities (Figures 7, 8, and S14–S17).

### Flow turbulence

Areas of increased turbulence (inferred from turbulent kinetic energy) were generated immediately downstream of the frondlets when the *Pectinifrons* models were oriented at 0° or 180° to the inlet (Figures 5, 6, and S7–S10). In these orientations, thin streaks of elevated turbulent kinetic energy were developed at the upper tips of all the frondlets (with the exception of the most marginal frondlets in the steep ‘U’-shaped model), with streaks extending downstream of the models and slightly upwards into the overlying water column. In contrast, at an orientation of 90° to the inlet, this zone of increased turbulence was largely restricted to the lateral frondlets (Figures 5, 6, and S7–S10). Because the one equation Spalart-Allmaras model does not explicitly solve the turbulent kinetic energy (*k*) magnitude parameter, it was not possible to visualize these patterns for the *C*. *lyra* models.

## Discussion

The CFD results allow us to test our original predictions surrounding hypothesized feeding modes in *Pectinifrons*, and also to compare and contrast patterns of flow with those reconstructed for the extant carnivorous sponge *C. lyra*. Below, we first discuss to what extent flow patterns support suspension feeding and osmotrophy in *Pectinifrons*, and subsequently explore whether the frondlets might represent adaptations for functions other than feeding. Finally, we discuss to what extent the similar fence-like morphologies of *Pectinifrons* and *C*. *lyra* represent convergent adaptations for common biological and/or ecological functions.

### Was *Pectinifrons* a suspension feeder?

We hypothesized that the interpretation of *Pectinifrons* as a sessile suspension feeder would be supported by: a) reconstructing parts of the anatomy extended into the water column as a collecting apparatus (or otherwise hosting structures that plausibly could have captured suspended food particles) that came into contact with large volumes of moving fluid; and/or b) the presence of recirculated flow toward putative feeding structures and/or localized areas of low-velocity and low-turbulence flow that would allow food particles to fall out of suspension.

In terms of the first criterion, *Pectinifrons* almost certainly lived with two rows of offset frondlets extended upwards, in some examples reaching >16 cm in height above the sediment-water interface,^11^^,^^51^^,^^52^ and thus well above the viscous sublayer.^53^ Moreover, our simulations clearly showed that the frondlets were extended into parts of the water column characterized by relatively high flow velocities (Figures 3, S3, and S4), and were thus situated at heights where they would intercept large volumes of moving fluid, consistent with a key prediction for suspension feeders. Set against this, however, is the observation that, despite ∼50 years of close scrutiny, there is (as yet) no evidence for any structures associated with rangeomorph frondlets—including in *Pectinifrons*—that could have plausibly been involved in capturing food particles.^1^^,^^9^^,^^15^ Although lensoid structures figured by Butterfield^10^ in a three-dimensionally preserved specimen of *Charnia* are suggestive of some small-scale internal morphology, similar structures have yet to be reported in any other published specimen (*Charnia* or otherwise), despite being considerably larger than other features typically preserved in Ediacaran fossils from Newfoundland. While this absence could conceivably be due to taphonomy, both the number of studies focused on rangeomorph morphology and taxonomy, and the preservation of fine-scale features on many fossil surfaces around the Avalon Peninsula^1^ render the taphonomic false absence of structures that could be used for suspension feeding as unlikely^33^ (although not impossible, given that some ostia can be as small as 2 microns in diameter). There is moreover no preserved evidence for musculature in *Pectinifrons* (or any rangeomorph^5^), and thus no evidence that the organism was capable of re-orienting itself to more directly intercept flow. A wide variety of modern suspension feeders that raise feeding organs up into the water column where they can more reliably intercept food particles are able to re-orient the attitude and angle of the feeding apparatus in response to shifting currents (for example, barnacles and stalked crinoids^29^). *Pectinifrons* frondlets were not attached laterally along the basal stolon and so were free to move independently; however, there is as yet no evidence that these were capable of being actively moved or rotated in a fashion that might aid in particle capture.

In terms of the second criterion, although some of our simulations showed the recirculation of low-velocity flow to parts of the models, these patterns were not consistent and varied considerably between morphotypes and orientations to current. For example, recirculation was evident when the models were oriented at 0° or 180° to the current (with flow in the wake directed back toward the frondlets in the very center of the model), but there was much less recirculation toward the lateral frondlets in these orientations and almost no recirculation when the models were oriented at 90° to the inlet (and even then, only to specific frondlets). Given that *Pectinifrons* was immobile and did not adopt a consistent orientation on the seafloor within the Mistaken Point Ecological Reserve, our results suggest that, unlike many other Ediacaran taxa interpreted as benthic suspension feeders,^22^^,^^23^^,^^24^^,^^27^ *Pectinifrons* was unlikely to be reliant on recirculated flow toward sites of particle capture for feeding.

One of the most unusual aspects of the reconstructed flow patterns around *Pectinifrons* relates to the recovered patterns of turbulent kinetic energy. Several Ediacaran taxa (in particular erniettomorphs^24^^,^^25^^,^^26^) have been shown to increase turbulence downstream of individuals at all orientations—a strategy thought to aid with gregarious suspension feeding in aggregated populations. However, patterns of turbulent flow in the wakes behind *Pectinifrons* frondlets varied significantly in our analyses, with the size, height, and direction (i.e., upwards into the water column vs. downwards toward the sediment surface) of turbulent flow changing with orientation to the current. This result suggests that, despite comprising a large proportion of two Mistaken Point communities,^51^ individual *Pectinifrons* would not have created flow conditions that would have facilitated suspension feeding by their neighbors within populations.

These combined observations illustrate the problems associated with reconstructing *Pectinifrons* as a suspension feeder. While the overall morphology of *Pectinifrons* is superficially consistent with interpretation as a macroscopic suspension feeder adapted for a sieving or direct interception method of particle capture, there remain outstanding questions surrounding where/how particles could have been captured. In addition, given the apparently random orientation of *Pectinifrons* on Mistaken Point bedding planes, our reconstructed flow patterns do not support the prediction that the fence-like morphology of this taxon consistently brought flow toward putative sites of particle capture, either individually, or as aggregated populations (for example, contrast the random orientation and flow patterns around *Pectinifrons* with corals in the order Alcyonacea^54^^,^^55^). We concede that this inference assumes a passive mode of suspension feeding, and does not account for (for example) the ciliary pumping mechanisms hypothesized by Butterfield.^10^ However, given the lack of compelling fossil evidence for the presence of any openings and/or compartments in the surface of rangeomorph fronds, we argue that the support for this model is, at best, equivocal.

### Was *Pectinifrons* an osmotroph?

We hypothesized that the interpretation of *Pectinifrons* as a macroscopic osmotroph would be supported if low velocity, low turbulence flow was distributed evenly over the surface of the organism (i.e., hydrodynamic characteristics that would help maximize the potential for nutrient absorption^21^). We do find evidence to suggest that the morphology of *Pectinifrons* was effective at creating local regions of low velocity and low turbulence flow, but these were unevenly distributed across the organism. In addition, although models in several orientations appear to have created areas of low velocity and low turbulence flow in their immediate wakes (see in particular our ‘S’-shaped models at 90° to current: Figures 3, 4, 5, 6, and S3–S10), and thus might have helped establish low-turbulence flow regimes that could have enabled the larger population to feed (i.e., gregarious osmotrophy), these patterns are very sensitive to the current direction. Also set against this are the observations that *Pectinifrons* populations are typically relatively low in density (∼3–8 individuals/m^2^)^51^^,^^56^ and spatially randomly distributed,^57^^,^^58^ suggesting that, even if the orientations were favorable, individuals were likely not aggregated sufficiently to take advantage of flow in each other’s wakes.

On smaller scales, we also hypothesized that inference of an osmotrophic feeding mode would be supported if the flow was distributed equally across the surface of the frondlets (see e.g., Singer et al.^37^). Our simulations instead show that flow was deflected either side of the upright frondlets and accelerated through the gaps between them, with little evidence for vertical flow up or down the surfaces of the frondlets where it might come into sustained contact with fractally patterned tissues (save for small areas at the extreme tips and bases; Figures S11–S13). We concede both that our models are rigid—and so do not deform in flow in the way that soft biological tissues would—and do not include the textured ‘fractal’ surfaces characteristic of rangeomorphs (which are expected to entrain fluid as it moved over the surface of the frondlets; see e.g. Singer et al.^37^). However, we note that there are also more theoretical problems at this scale—Butterfield^10^ points out that the external digestion of labile dissolved organic carbon is significantly inhibited at higher Reynolds number regimes (such as those typically experienced by macroscopic organisms) because advective nutrient loss vastly outpaces viscous entrainment, and thus inhibits external digestion and nutrient transport across tissues.^59^^,^^60^^,^^61^ In addition, the size of the Neoproterozoic DOC pool is now thought to have been comparable to the present day,^62^ presenting substantial barriers to supporting macroscopic body sizes while feeding via osmotrophy. Thus, while we note that the reconstructed flow patterns around *Pectinifrons* are unique in comparison to all other Ediacaran taxa studied with CFD,^21^^,^^22^^,^^23^^,^^24^^,^^25^^,^^26^^,^^27^ they are not easily allied with an osmotrophic feeding habit either.

### Insights from comparison with *C*. *lyra*

Comparing simulated flow patterns between *Pectinifrons* and *C. lyra* reveals both striking similarities, and differences, between the two taxa. In terms of similarities, flow accelerates between adjacent vertical branches in *C*. *lyra*, as it does between the raised frondlets of *Pectinifrons*. However, the principal difference between the two models hinges on the far lower levels of recirculation seen behind *C. lyra*; plots of negative streamwise velocity (Figures 8, S16, and S17) show some recirculation behind the basal stolons close to the sediment-water interface, but very little behind upright branches (much less than is seen behind *Pectinifrons* frondlets at certain orientations; Figures 4, S5, and S6). The greater levels of recirculation seen behind *Pectinifrons* are almost certainly due to the relatively wide frondlets—which present a greater cross-sectional area to flow (and thus produce larger wakes)—in comparison to the relatively thin branches of *C*. *lyra*. Consequently, these patterns are sensitive to our choice of reconstruction. On the basis of the fossil evidence, we do, however, think it overwhelmingly likely that *Pectinfrons* frondlets were flattened parallel to the axis of the basal stalk (i.e. with elliptical cross-sections), and thus that these flow patterns are realistic. We concede that, given that adjacent frondlets were not attached laterally, it is possible that they would have been affected by flow independently. Thus, while there is no evidence that *Pectinifrons* was capable of actively moving or re-orienting individual frondlets, frondlets may have rotated passively, potentially having a variety of effects on patterns of flow and recirculation. However, given the computational challenges associated with simulating fluid-structure interactions, we are currently unable to test this model.

We suggest that the differences in modeled flow patterns around *Pectinifrons* and *C*. *lyra* reflect different functional morphologies. The thin and cylindrical upright branches possessed by *C. lyra* are readily interpretable as an adaptation to minimize drag, and thus encourage a steady flow between branches that bring suspended prey items that can be caught and consumed (and without interference from recirculating flow that might dislodge captured organisms). In this fashion, the morphology of *C*. *lyra* can be interpreted as functioning as a macroscopic filtering array, employing a combination of sieving and direct interception strategies for particle capture (*sensu* LaBarbera^40^). This strategy also helps to emphasize the difficulty in interpreting the morphology of *Pectinifrons* in terms of adaptations to feeding; our reconstructed flow patterns illustrate that—given the differences in flow patterns with changing orientation—not only is it unlikely that food particles were being captured on the surfaces of frondlets, it is also unlikely that particles were somehow being caught between them. In sum, therefore, our results suggest that *Pectinifrons* and extant harp sponges represent an unusual case of morphological convergence on a fence-like morphology, but with different hydrodynamic consequences, and associated with different biological and/or ecological functions.

### Rangeomorph fronds as respiratory organs

Thus far, we have only considered reconstructed flow patterns around *Pectinifrons* frondlets as potential adaptations for feeding. However, an alternative scenario is that rangeomorph elements were instead organs adapted for respiration and gas exchange. Ghisalberti et al.^63^ noted that the faster fluid velocities associated with rangeomorph taxa occupying at greater heights above the sediment-water interface could have favored the absorption of oxygen. Moreover, Singer et al.^37^—using models in flume-tanks—showed that fronds would likely have oscillated or vibrated in flow; this behavior would not have been advantageous for filter feeding,^64^ but could plausibly have enhanced gas exchange. In this light, the observed acceleration of fluid between rows of frondlets might have served to reduce the thickness of the diffusive boundary layer adjacent to frond surfaces (and thus enhance the transmission of gasses across membranes), while the fractal organization of the frond itself would provide a high SA/V with which to conduct gas exchange. This model is also consistent with several other facets of the hydrodynamics and paleoecology of *Pectinifrons*. For example, gas exchange would be facilitated by high velocity and low turbulence flow regimes, such that the ‘dead zone’ created behind the front row of frondlets would help the rest of the organism uptake oxygen and dispose of waste gases.

Interestingly, a prediction of this model might be that *Pectinifrons* frondlets would have oscillated or vibrated in flow similar to what has been observed in physical experiments by Singer et al.^37^ This predicted response would scale with tissue rigidity, whereby a more labile frondlet could deform and/or oscillate with ease, and more rigid frondlet bodies would more likely develop vortex-induced vibrations (VIVs). VIVs occur when the boundary layer surrounding an object is forced to separate due to the object’s curvature, thus shedding as a vortex by changing the pressure distribution.^65^ Due to asymmetric vortex shedding from unidirectional flow, the object begins to vibrate. Regardless of vibration or oscillation intensity, these flow features would impede suspension feeding, and thus constitute more evidence for gas exchange.^64^ Emerging and more computationally complex techniques in modeling fluid-structure interaction (‘FSI’; coupled CFD and computational structural mechanics) may offer a means of testing this hypothesis in the future.^21^ In particular, analysis of fluid flow at much smaller scales—incorporating the fractal surfaces of frondlets (something not possible in the simulations performed here)—would be an invaluable step toward determining whether the increased surface area and roughness associated with fractal tissues vibrating in the water column may have played a greater role in increasing rates of gas exchange, or in creating slow-moving, linear fluid flow over tissues that would favor osmotrophy (or conceivably both). Further incorporating a diffusive species component, and a prescribed flux across the frond surface, would improve future models addressing these outstanding questions. On the basis of theory and past fluid dynamics work, we predict increased viscous sublayer heights in the fractal surface regions, that would then compress and expand during the oscillation of the overall body of the frond in flow. In a gas exchange scenario, we propose that this would likely correspond with increased and decreased gas concentration gradients between the far field flow and the immediate vicinity of the tissue surface.

Reconstructing rangeomorph elements as structures designed to maximize gas exchange, rather than feeding, would require a dramatic re-thinking of rangeomorph paleobiology (see e.g., Burzynski et al.^66^). Rather than organisms with morphologies adapted to maximize the osmotic uptake of dissolved organic carbon,^51^^,^^63^^,^^67^ this model would instead re-imagine rangeomorphs as adapted to scavenging oxygen in one of the few Avalon-aged marine environments where conditions allowed macroscopic organisms to thrive (i.e., the ‘deep-marine stenothermal cradle of Ediacaran evolution’ of Boag et al.^68^). This hypothesis may also align with several other unusual aspects of Mistaken Point community dynamics. It could, for example, help explain why the Mistaken Point communities had different ecological dynamics to modern marine communities.^56^^,^^58^ The morphology of many rangeomorph genera may have evolved to take advantage of oxygenated water currents produced by the complex 3D flow fields generated by *in situ* benthic communities. Although speculative, this is a hypothesis that could be tested via larger scale CFD analyses.

This model does, however, leave the question as to how *Pectinifrons* (and other rangeomorphs) obtained nutrients open. Our reconstructed flow patterns do not offer strong support for—but cannot strictly reject—the hypothesis that *Pectinifrons* was a suspension feeder. Equally, flow patterns are not strictly consistent with osmotrophy. Reconstructing rangeomorph fronds as organs adapted for respiration and gas exchange does not necessarily preclude them from performing other functions, but on the basis of our data, there is little evidence to favor either of these two hypothesized feeding modes. Moreover, the deep-water paleoenvironment at Mistaken Point in which these organisms are found precludes photoautotrophy.^15^ One other possibility is chemoautotrophy—an idea recently championed by McIlroy et al.^31^^,^^69^—which proposes that rangeomorph tissues possessed sulfur-oxidizing ectosymbionts, that in turn took advantage of sulfidic porewaters generated in the sediment underneath organisms. Although many of the reconstructions favored by these authors apparently require frondose rangeomorphs to be reclining on the seafloor—something at odds with the majority of fossil evidence^12^^,^^13^^,^^32^^,^^51^^,^^70^—our study does suggest that chemoautotrophy requires more detailed testing. It has been hypothesized that the holdfasts of some rangeomorphs may have played a role in nutrient acquisition,^66^^,^^71^ a suggestion that may ultimately be more plausible, but would require both new anatomical and geochemical data to adequately test.

### Limitations of the study

Our analyses incorporate several simplifications that could have influenced the results. In particular, the digital models of *P*. *abyssalis* and *C*. *lyra* lack fine anatomical details, such as the fractal surfaces of the frond in the former and the rows of filaments arising from vertical branches in the latter, which might have affected flow patterns. Similarly, the seafloor was represented as a simple smooth surface rather than as a more realistic textured one. Lastly, we used rigid models that did not deform as fluid moved past them, but it is possible that the living organisms would have oscillated or vibrated in flow. These simplifications were necessary owing to computational limitations. As computer power increases, future work simulating fluid-structure interactions with more detailed digital models will serve as a test of our findings.

### Conclusions

CFD simulations around models of the fence-like Ediacaran rangeomorph *Pectinifons abyssalis* produced unique flow patterns that are unlike those seen for any other Ediacaran taxon. Moreover, comparisons with the morphologically similar extant carnivorous sponge *C*. *lyra* revealed significant differences between the two organisms, indicating that macroscopic eukaryotes separated by ∼500 million years converged on similar fence-like forms, but for different biological and/or ecological reasons. Although there are potential scale-related shortfalls in our CFD analyses, we argue that the modeled flow patterns around *Pectinifrons* do not provide support for either a suspension feeding or osmotrophic feeding habit. While the overall morphology of *Pectinifrons* is consistent with interpretation as a macroscopic suspension feeder, both the sensitivity of flow patterns to orientation on the bedding plane and the lack of preserved structures that could have been involved in particle capture highlight problems with this reconstruction. On the basis of these CFD simulations, the feeding mode of *Pectinifrons* thus remains unresolved. However, we suggest that some aspects of the reconstructed flow patterns—specifically, the presence of relatively high velocity and low turbulence flow in-between rows of frondlets—support the suggestion that rangeomorph fronds were organs adapted for oxygen uptake and gas exchange, rather than feeding. This interpretation provides several testable predictions with respect to the behavior of fronds in flow and—if supported—would represent a dramatic reinterpretation of the paleobiology of rangeomorphs, and thus potentially the earliest macroscopic animals.

## STAR★Methods

### Key resources table

**Table:** 

REAGENT or RESOURCE	SOURCE	IDENTIFIER
**Deposited data**		

Digital models	This paper	https://vanderbilt365.sharepoint.com/:f:/s/Ediacaranfluiddynamics/EpSiIbn8B6RCvFbRqOcL1XgBqfElZdRZbUMG2qywaBBC9g
CFD results files	This paper	https://vanderbilt365.sharepoint.com/:f:/s/Ediacaranfluiddynamics/EpSiIbn8B6RCvFbRqOcL1XgBqfElZdRZbUMG2qywaBBC9g

**Software and algorithms**		

Rhinoceros 3D version 7	Robert McNeel & Associates	https://www.rhino3d.com
COMSOL Multiphysics version 6	COMSOL, Inc.	https://www.comsol.com

### Resource availability

#### Lead contact

Further information and requests for resources should be directed to and will be fulfilled by the lead contact, Imran Rahman (imran.rahman@nhm.ac.uk).

#### Materials availability

This study did not generate new materials.

### Experimental model and subject details

#### Material

*P. abyssalis* is known from the Mistaken Point and Trepassey formations in the Avalon Peninsula, Newfoundland, Canada. Fossils are preserved as molds under beds of volcanic ash (“Conception-style” preservation of Narbonne^72^). The taphonomic pathway inferred for soft-tissue preservation in these fossils is similar to the microbial ‘‘death mask’’ originally proposed by Gehling,^73^ which is supported by the presence of framboidal pyrite veneers on fossiliferous surfaces.^74^^,^^75^ Most fossil-bearing horizons are interpreted as comprising turbidite beds deposited in a continental slope setting at >200 m water depth, in an environment influenced by contour currents.^12^^,^^13^ Recent geochronological analysis of ash beds preserved around the Avalon Peninsula date fossil occurrences to the interval spanning 574–564 Ma, with *Pectinifrons* constrained to the upper Briscal through lower Fermeuse formations (∼567–563 Ma^76^).

### Method details

#### Digital modeling

Three-dimensional digital models of *Pectinifrons* and *C. lyra* were created using the 3D computer graphics and computer-aided design software Rhinoceros 3D version 7 (https://www.rhino3d.com). Specimen photographs, line drawings and life reconstructions^11^^,^^18^ were imported into the program as background images and scaled to their original size. Models were then constructed using a non-uniform rational basis spline (NURBS) approach. Stalks and frondlets (*Pectinifrons*) and stolons and vertical branches (*C*. *lyra*) were created from ellipsoids, and these were then scaled to match the dimensions reported in Bamforth et al.^11^ and Lee et al.,^18^ respectively. In each model, all objects were joined using the Boolean Union function. We reconstructed three *Pectinifrons* models based on the different stalk morphologies (gentle ‘U’-shaped, steep ‘U’-shaped and ‘S’-shaped) known from Mistaken Point (Figures 1, Figures 2A–2C). In addition, we created models of *C*. *lyra* with three and two vanes based on the morphology of the holotype and the paratype, respectively (Figures 2D and S1). Digital models are available from: https://vanderbilt365.sharepoint.com/:f:/s/Ediacaranfluiddynamics/EpSiIbn8B6RCvFbRqOcL1XgBqfElZdRZbUMG2qywaBBC9g.

#### Computational fluid dynamics

Computational fluid dynamics (CFD) analyses were performed using the simulation software COMSOL Multiphysics version 6 (https://www.comsol.com), following protocols outlined in Gibson et al.^21^ The computational domain (Figure 2E) consisted of a cuboid measuring 7.32 m in length, 5.75 m in width and 0.60 m in height. Models were centrally placed on the lower boundary of the domain such that the cuboid extended at least 3× the length of the model upstream, 10× the length of the model downstream, and 5× the size of the model in all other directions. A Boolean operation was used to subtract the model from the domain, and the material properties of water were then assigned to the space surrounding the model. An inlet with a fully developed flow (depth-averaged velocity) was specified at one end of the domain and an outlet with a static pressure equal to 0 Pa was defined at the opposing end. No-slip boundaries were assigned to the model and the bottom of the domain, with slip boundaries used for the top and sides of the domain. The domain was meshed using a region of prismatic elements along the no-slip boundaries, with tetrahedral elements used in the rest of the domain. A refinement area consisting of a second cuboid measuring at least 2× the length and width of the model and 1.5× the height of the model was used to create a much finer mesh in parts of the domain close to the model (Figures 2F and 2G). A sensitivity analysis was carried out using the gentle ‘U’-shaped *Pectinifrons* model to determine the coarsest mesh at which the results (i.e., plots of flow velocity magnitude, *U*) were independent of the mesh size (Figure S2), and this was then selected for use in all subsequent simulations. The Reynolds-averaged Navier–Stokes (RANS) equations were solved using the two-equation shear-stress transport (SST) turbulence model for the *Pectinifrons* models and the simpler one-equation Spalart-Allmaras turbulence model for the *C*. *lyra* models. The Spalart-Allmaras closure was necessary in the latter case to improve convergence and economize on computational resources due to the inherently greater number of mesh elements required to accurately reconstruct flow around *C. lyra*. Both sets of equations simulate flow in the turbulent regime, consistent with the benthic boundary layer, which is naturally turbulent even at low velocities. In all these simulations, a stationary solver was used to obtain a solution approximating time-averaged patterns.

We simulated inlet velocities of 0.05, 0.1, and 0.2 m/s for each model. These values were selected because they reflect the typical range of current speeds recorded from modern deep water (i.e., continental slope) settings^77^ similar to those inhabited by *Pectinifrons* in the Ediacaran^12^^,^^13^ and present-day *C*. *lyra*.^18^ We also carried out simulations with the models at different orientations to the inlet: 0°, 90°, and 180° for the two ‘U’-shaped *Pectinifrons* models and the three-vaned *C*. *lyra* model; and 0° and 90° for the ‘S’-shaped *Pectinifrons* model and the two-vaned *C*. *lyra* model (geometries identical for orientations of 0° and 180°). This was done to account for the apparently random orientations exhibited by *Pectinifrons* specimens on bedding surfaces^11^^,^^32^ and uncertainty regarding the preferred orientation of *C*. *lyra* specimens to flow.

### Quantification and statistical analysis

Using COMSOL Multiphysics, we visualized CFD results as two-dimensional plots (horizontal and vertical cross-sections) of velocity magnitude (*U*) with flow vectors, velocity component *w* (vertical component of velocity), and *k* (turbulent kinetic energy; *Pectinifrons* only). In addition, we exported three-dimensional isosurface plots of negative values of velocity component *u* (streamwise velocity) and *k* (turbulent kinetic energy; *Pectinifrons* only). CFD results files are available from: https://vanderbilt365.sharepoint.com/:f:/s/Ediacaranfluiddynamics/EpSiIbn8B6RCvFbRqOcL1XgBqfElZdRZbUMG2qywaBBC9g.

## Data Availability

• Digital models and CFD results files have been deposited at SharePoint (https://vanderbilt365.sharepoint.com/:f:/s/Ediacaranfluiddynamics/EpSiIbn8B6RCvFbRqOcL1XgBqfElZdRZbUMG2qywaBBC9g) and are publicly available as of the date of publication. • This paper does not report original code. • Any additional information required to reanalyze the data reported in this paper is available from the lead contact upon request.
