# Supplementary material for: The rangeomorph Pectinifrons abyssalis: Hydrodynamic function at the dawn of animal life
Source: iScience. 2023 Jan 16;26(2):105989. doi: 10.1016/j.isci.2023.105989 (PMC9900436; doi:10.1016/j.isci.2023.105989)
Supplement: Document S1. Figures S1–S20 [file mmc1.pdf]

iScience, Volume *26*

Supplemental information

The rangeomorph *Pectinifrons abyssalis*: Hydrodynamic function at the dawn of animal life

Simon A.F. Darroch, Susana Gutarra, Hale Masaki, Andrei Olaru, Brandt M. Gibson, Frances S. Dunn, Emily G. Mitchell, Rachel A. Racicot, Gregory Burzynski, and Imran A. Rahman

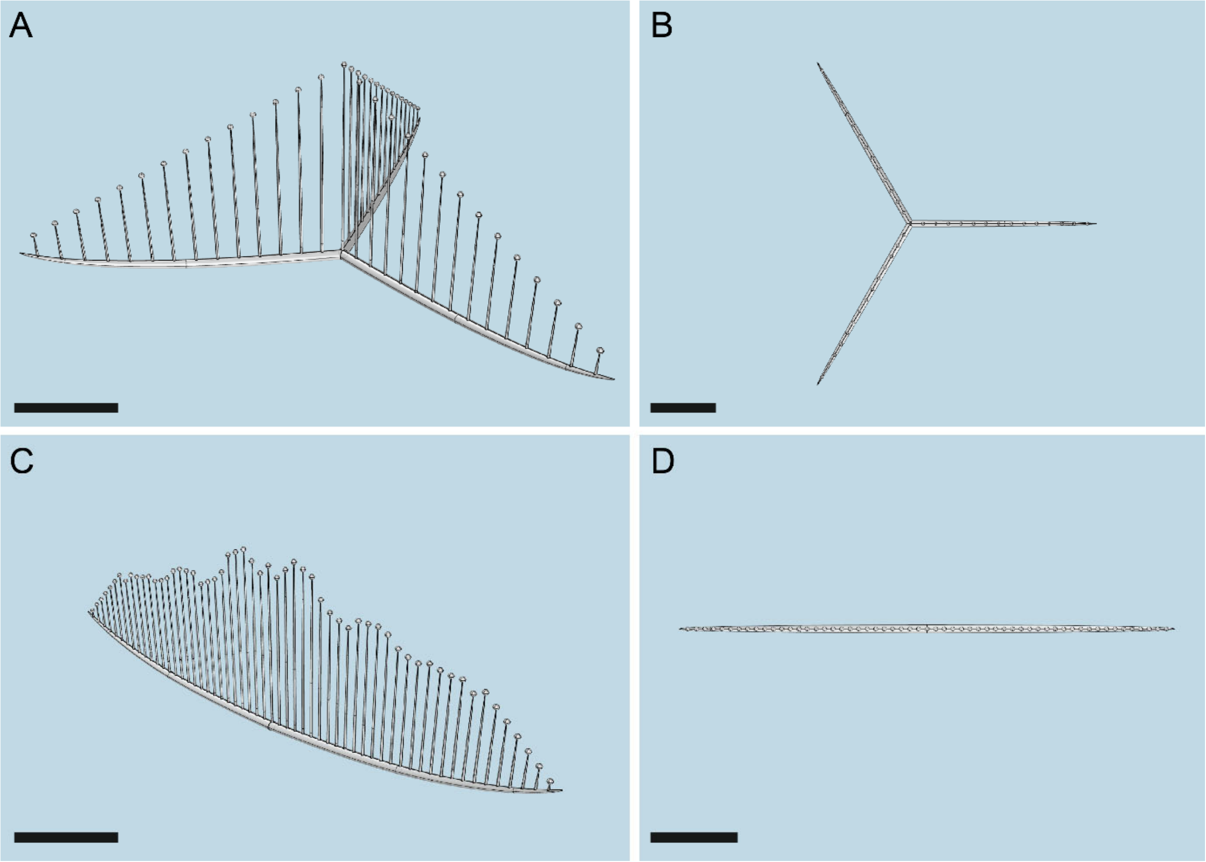

Figure S1. Digital models of *Chondrocladia lyra*, related to Figure 2. (A, B) Digital models of three-vaned *C. lyra* in angled (A) and upper (B) views. (C, D) Digital models of two-vaned *C. lyra* in angled (C) and upper (D) views. Scale bars: 10 cm.

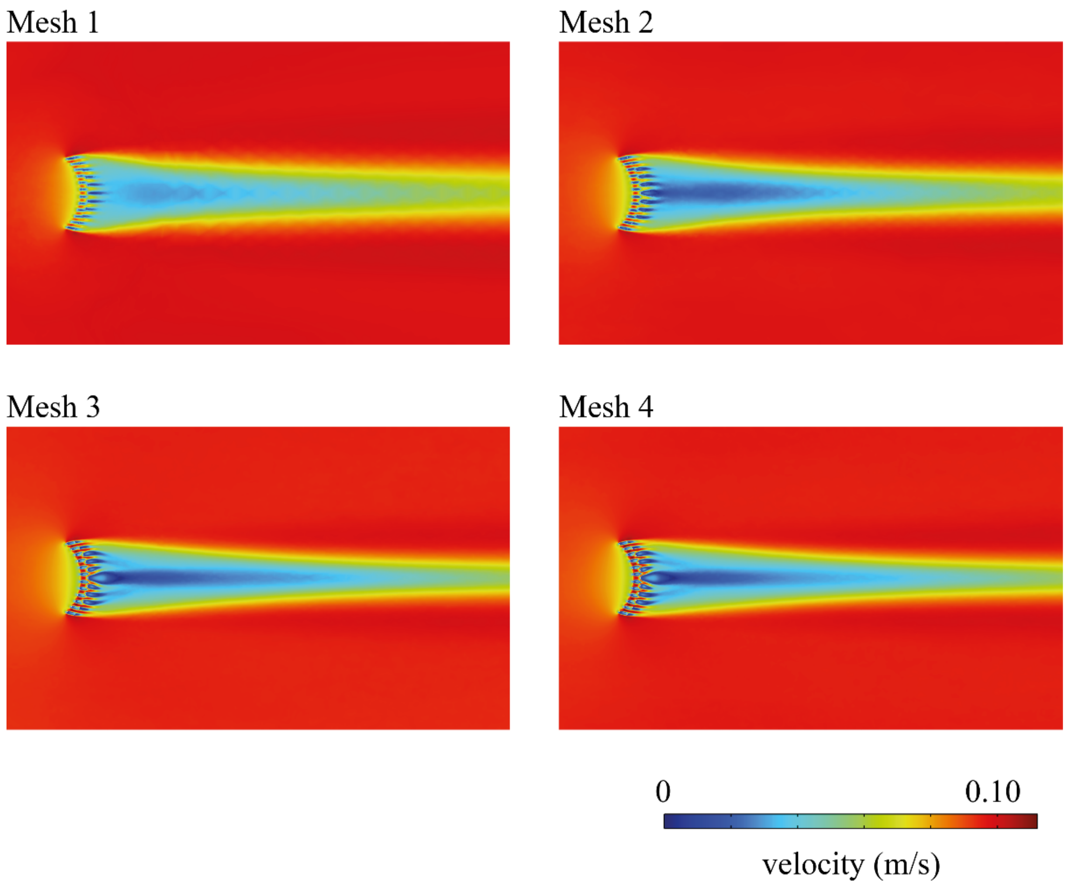

Figure S2. Mesh sensitivity analysis, related to Figure 2. Two-dimensional plots (horizontal cross-sections) of velocity magnitude (*U*) at an inlet velocity of 0.1 m/s for four increasingly fine meshes (A–D). (A) Mesh 1, ~700,000 mesh elements. (B) Mesh 2, ~1,600,000 mesh elements. (C) Mesh 3, ~4,900,000 mesh elements. (D) Mesh 4, ~7,200,000 mesh elements. Mesh 3 was selected for use in all subsequent simulations. Direction of ambient flow from left to right.

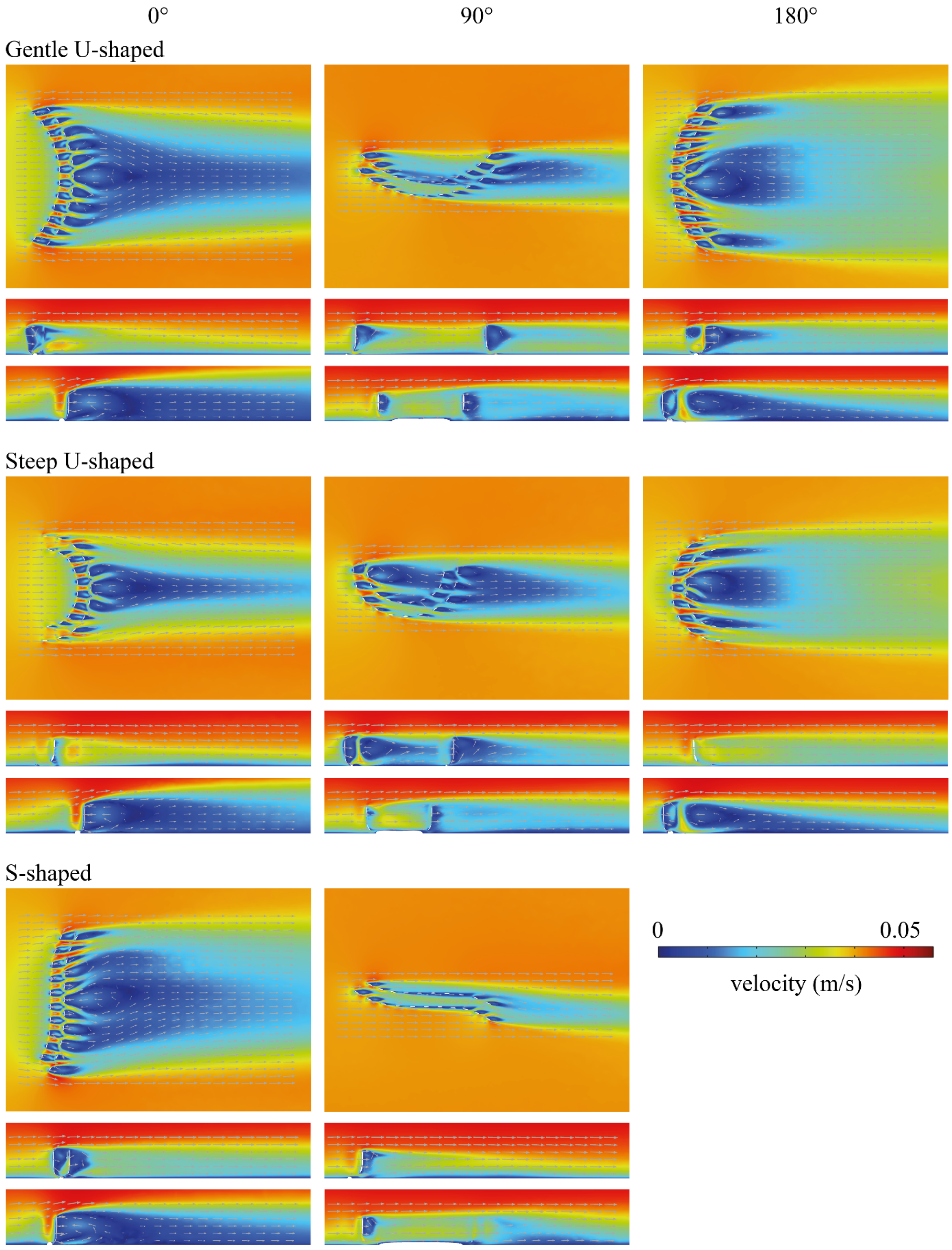

Figure S3. Two-dimensional plots (horizontal and vertical cross-sections) of velocity magnitude (*U*) with flow vectors (size of grey arrows proportional to natural logarithm of velocity magnitude) at an inlet velocity of 0.05 m/s for three *Pectinifrons* models, related to Figure 3. Direction of ambient flow from left to right.

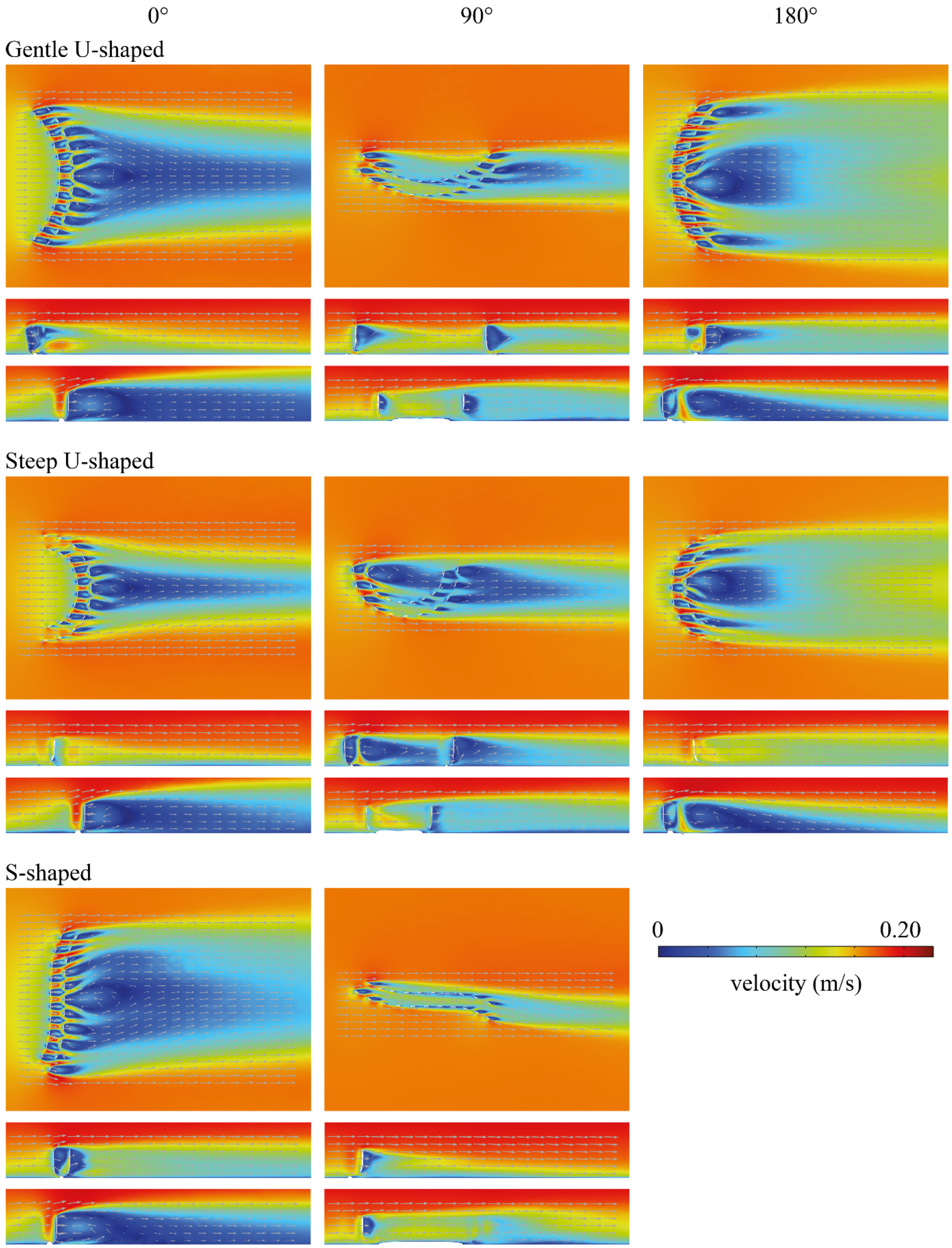

Figure S4. Two-dimensional plots (horizontal and vertical cross-sections) of velocity magnitude (U) with flow vectors (size of grey arrows proportional to natural logarithm of velocity magnitude) at an inlet velocity of 0.2 m/s for three *Pectinifrons* models, related to Figure 3. Direction of ambient flow from left to right.

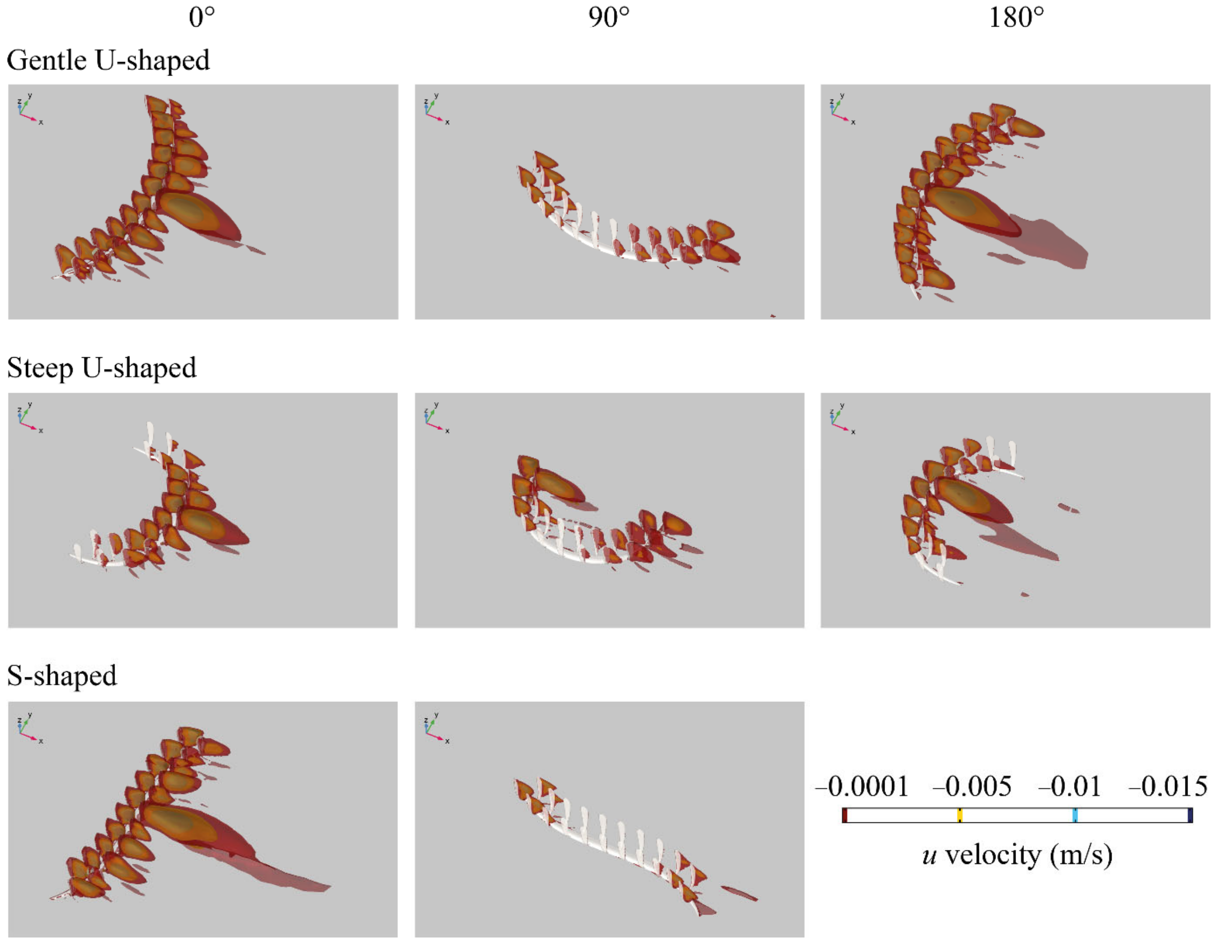

Figure S5. Three-dimensional isosurface plots of negative values of velocity component *u* (streamwise velocity) at an inlet velocity of 0.05 m/s for three *Pectinifrons* models, related to Figure 4. Direction of ambient flow from top left to bottom right.

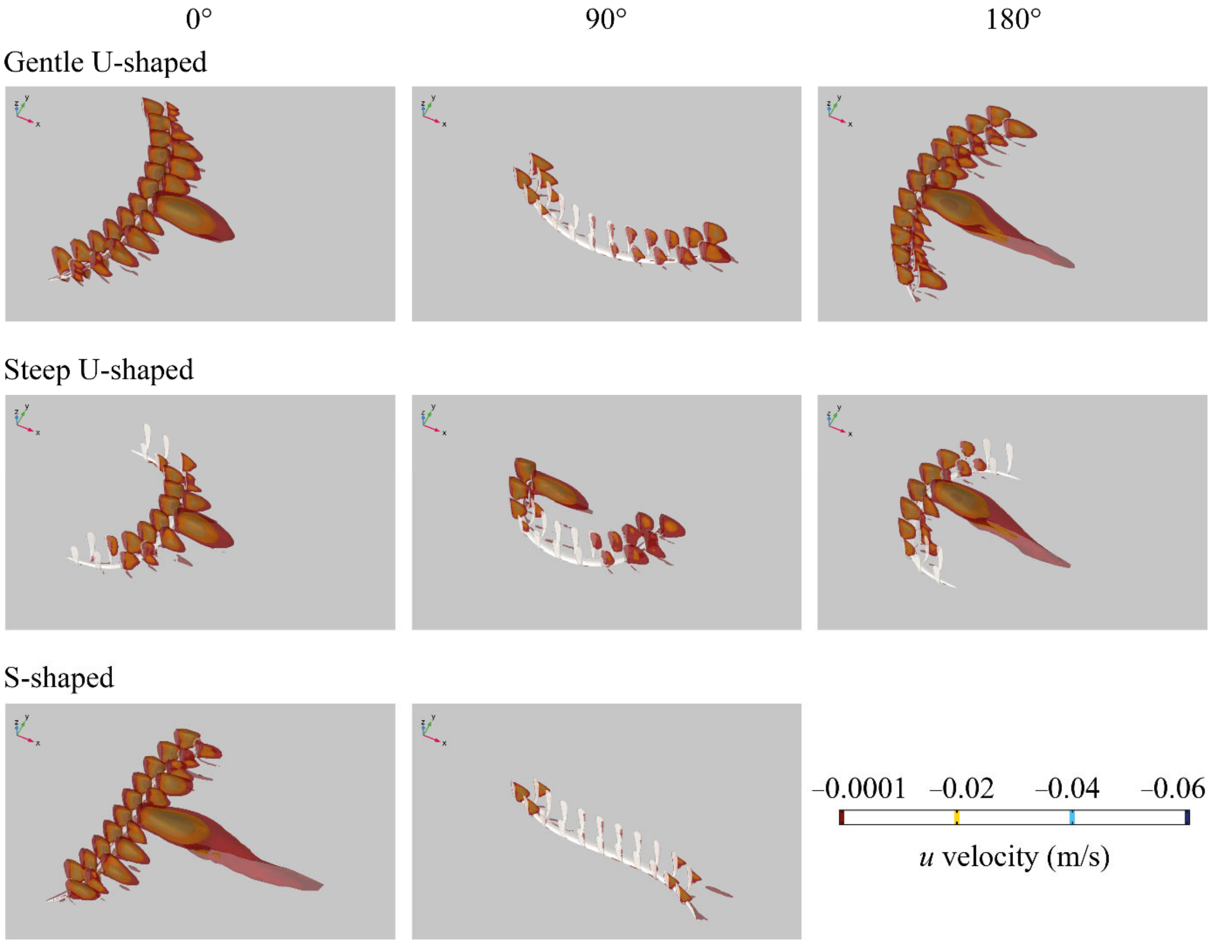

Figure S6. Three-dimensional isosurface plots of negative values of velocity component *u* (streamwise velocity) at an inlet velocity of 0.2 m/s for three *Pectinifrons* models, related to Figure 4. Direction of ambient flow from top left to bottom right.

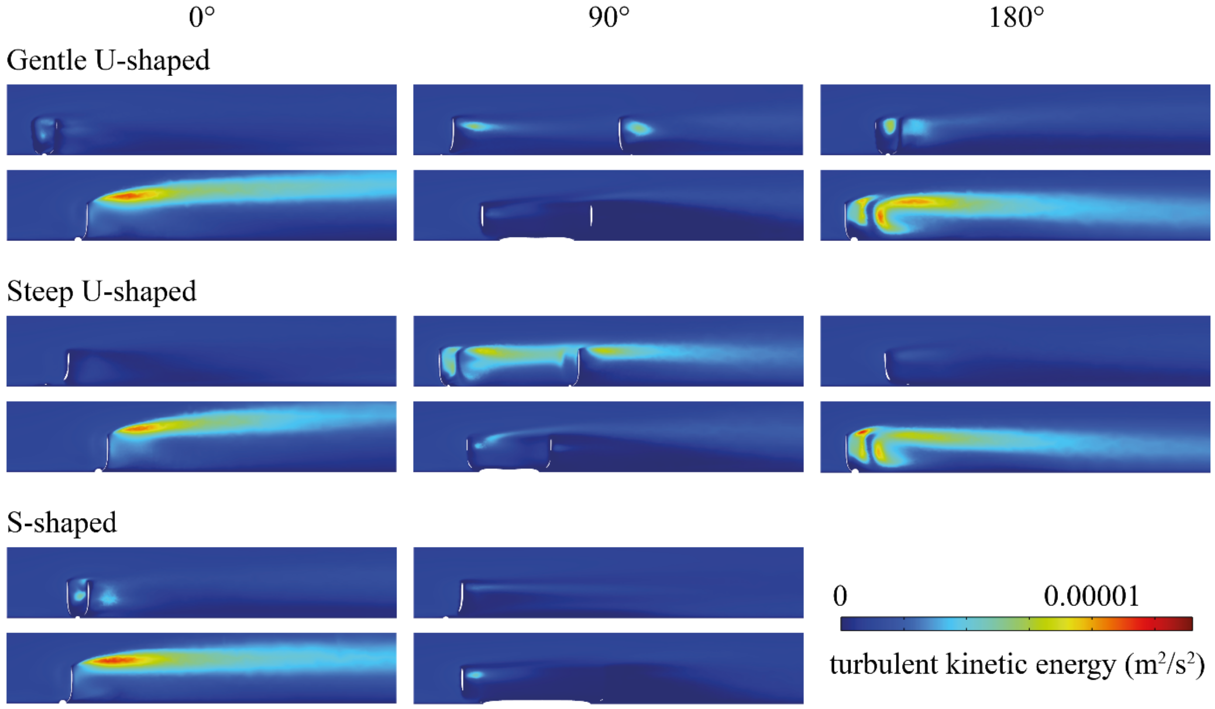

Figure S7. Two-dimensional plots (vertical cross-sections) of turbulent kinetic energy magnitude (*k*) at an inlet velocity of 0.05 m/s for three *Pectinifrons* models, related to Figure 5. Direction of ambient flow from left to right.

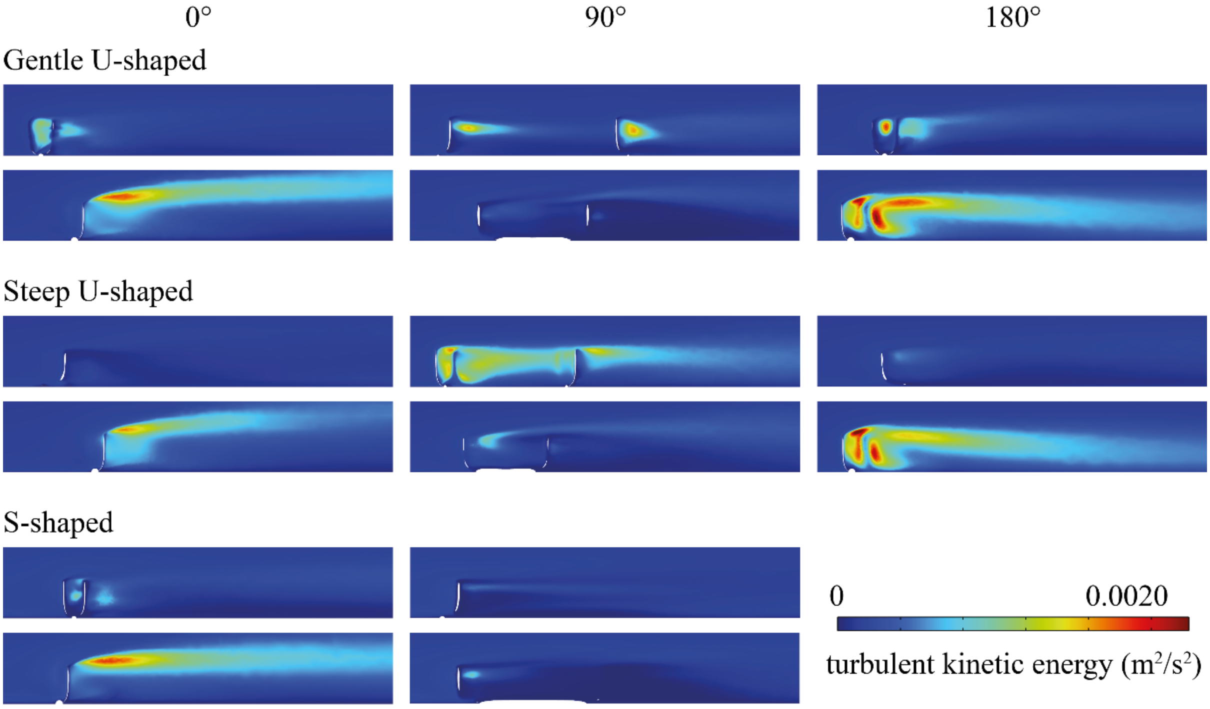

Figure S8. Two-dimensional plots (vertical cross-sections) of turbulent kinetic energy magnitude (*k*) at an inlet velocity of 0.2 m/s for three *Pectinifrons* models, related to Figure 5. Direction of ambient flow from left to right.

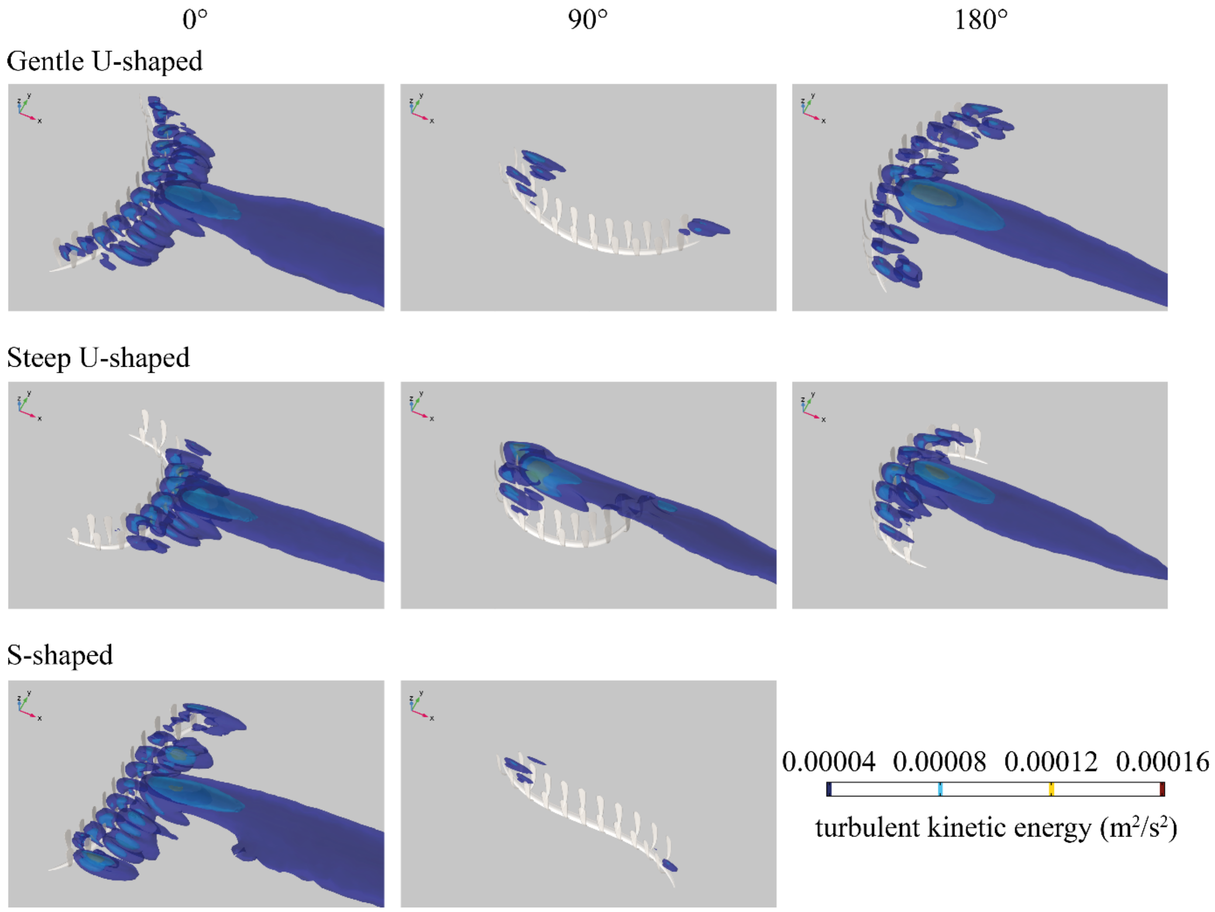

Figure S9. Three-dimensional isosurface plots of turbulent kinetic energy magnitude (*k*) at an inlet velocity of 0.05 m/s for three *Pectinifrons* models, related to Figure 6. Direction of ambient flow from top left to bottom right.

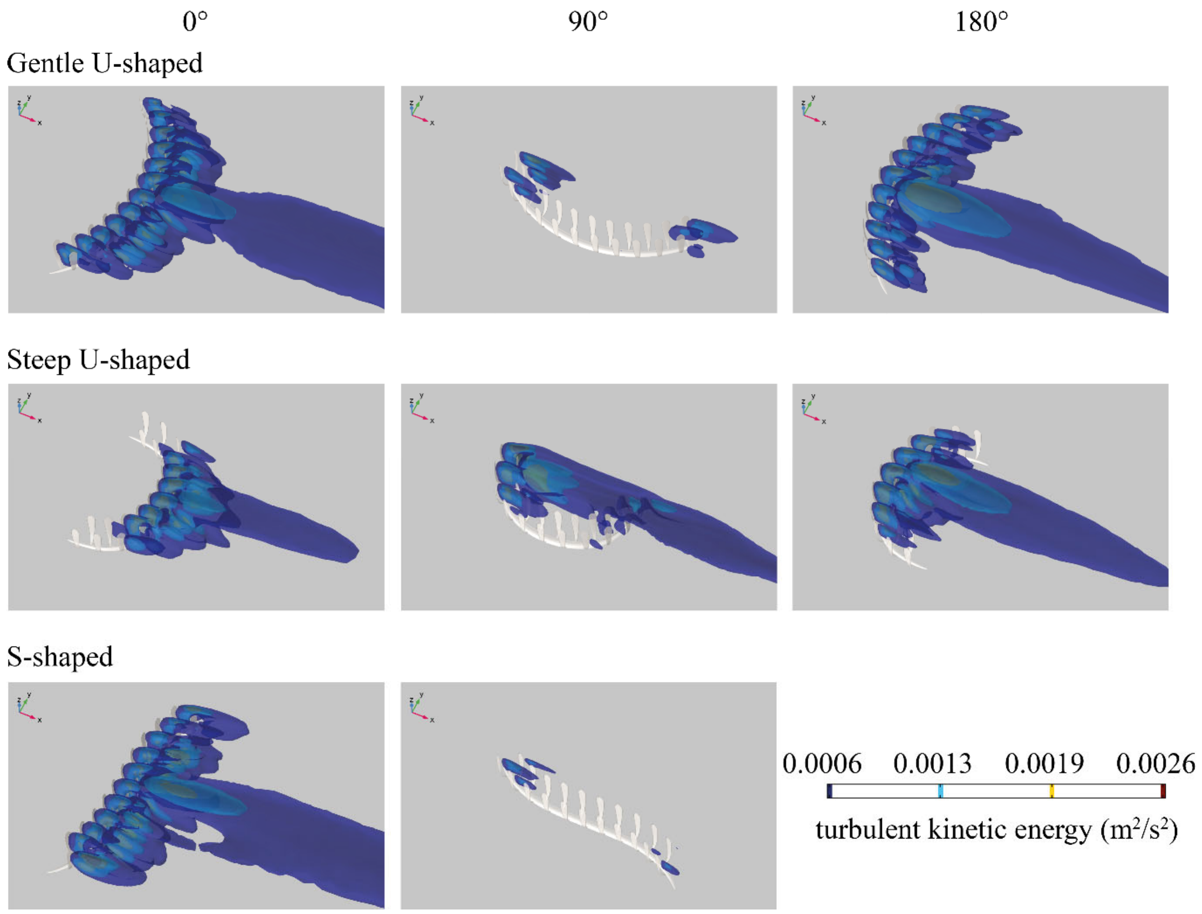

Figure S10. Three-dimensional isosurface plots of turbulent kinetic energy magnitude (k) at an inlet velocity of 0.2 m/s for three *Pectinifrons* models, related to Figure 6. Direction of ambient flow from top left to bottom right.

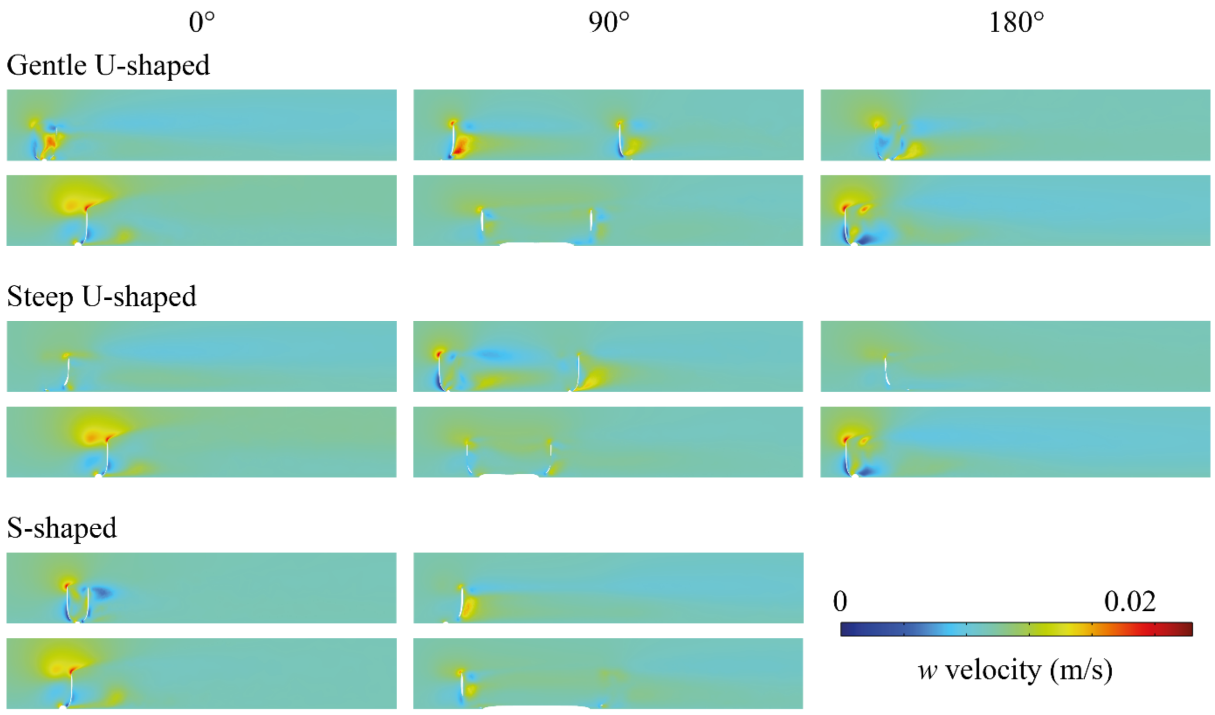

Figure S11. Two-dimensional plots (vertical cross-sections) of velocity component *w* (vertical velocity) at an inlet velocity of 0.05 m/s for three *Pectinifrons* models, related to Figure 3. Direction of ambient flow from left to right.

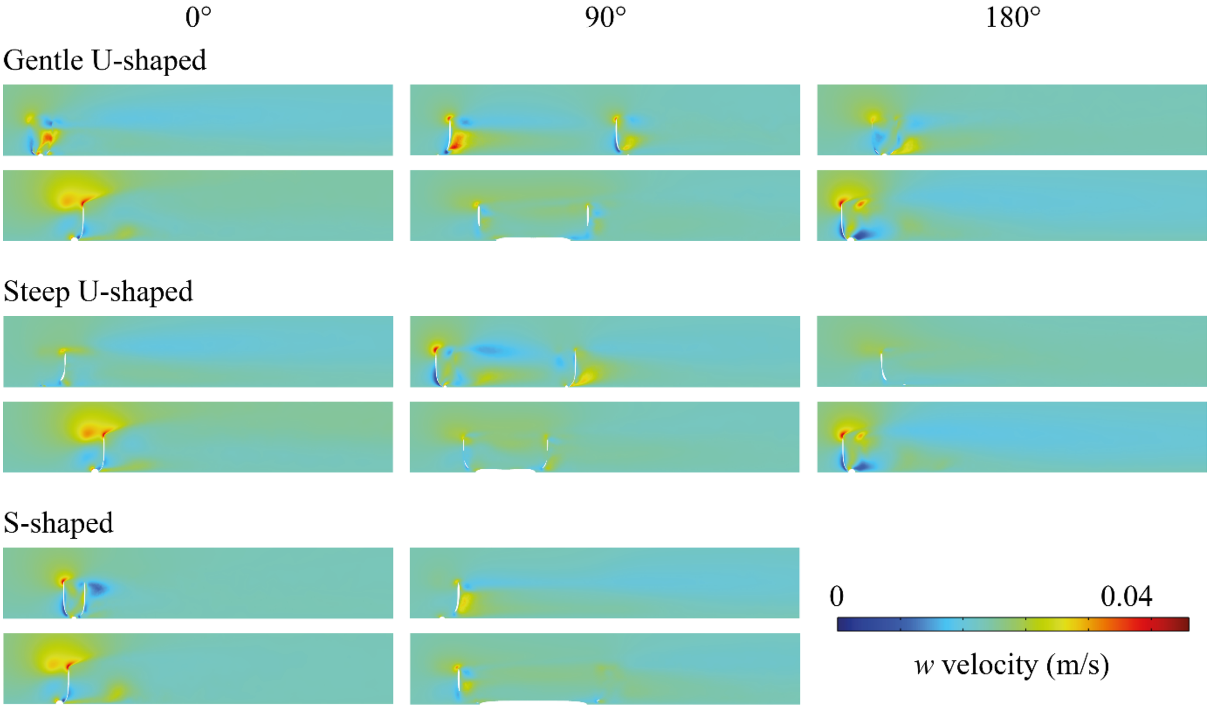

Figure S12. Two-dimensional plots (vertical cross-sections) of velocity component *w* (vertical velocity) at an inlet velocity of 0.1 m/s for three *Pectinifrons* models, related to Figure 3. Direction of ambient flow from left to right.

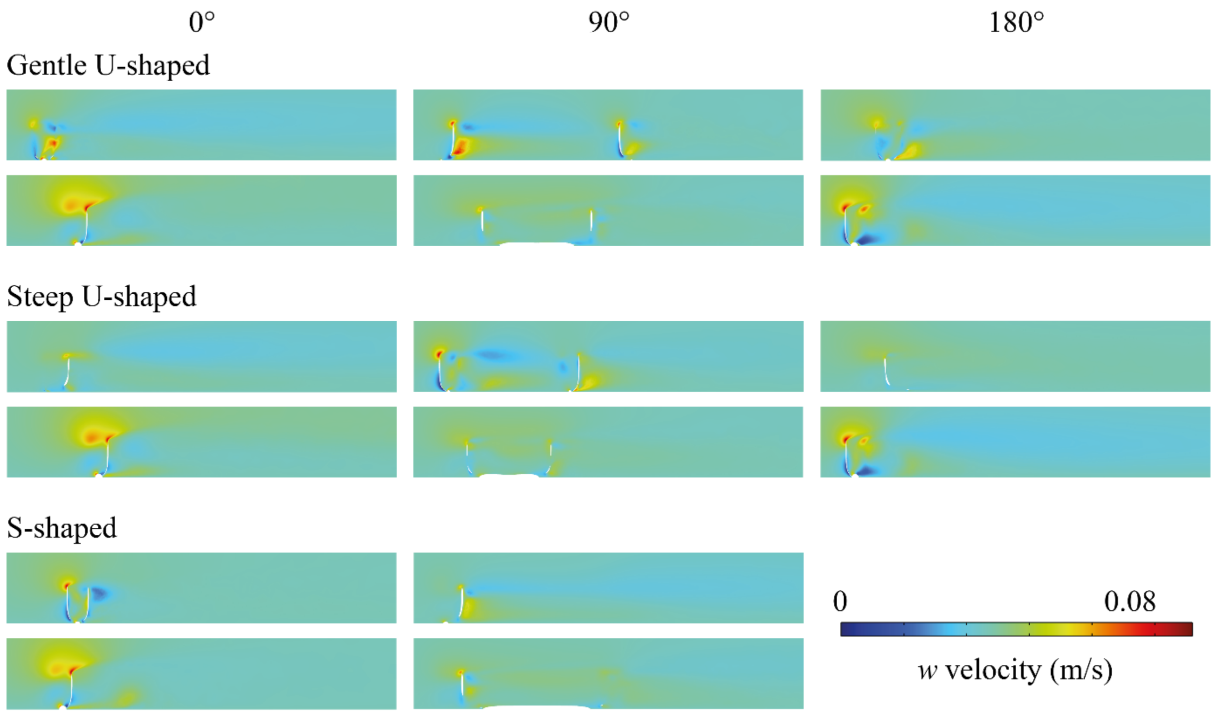

Figure S13. Two-dimensional plots (vertical cross-sections) of velocity component *w* (vertical velocity) at an inlet velocity of 0.2 m/s for three *Pectinifrons* models, related to Figure 3. Direction of ambient flow from left to right.

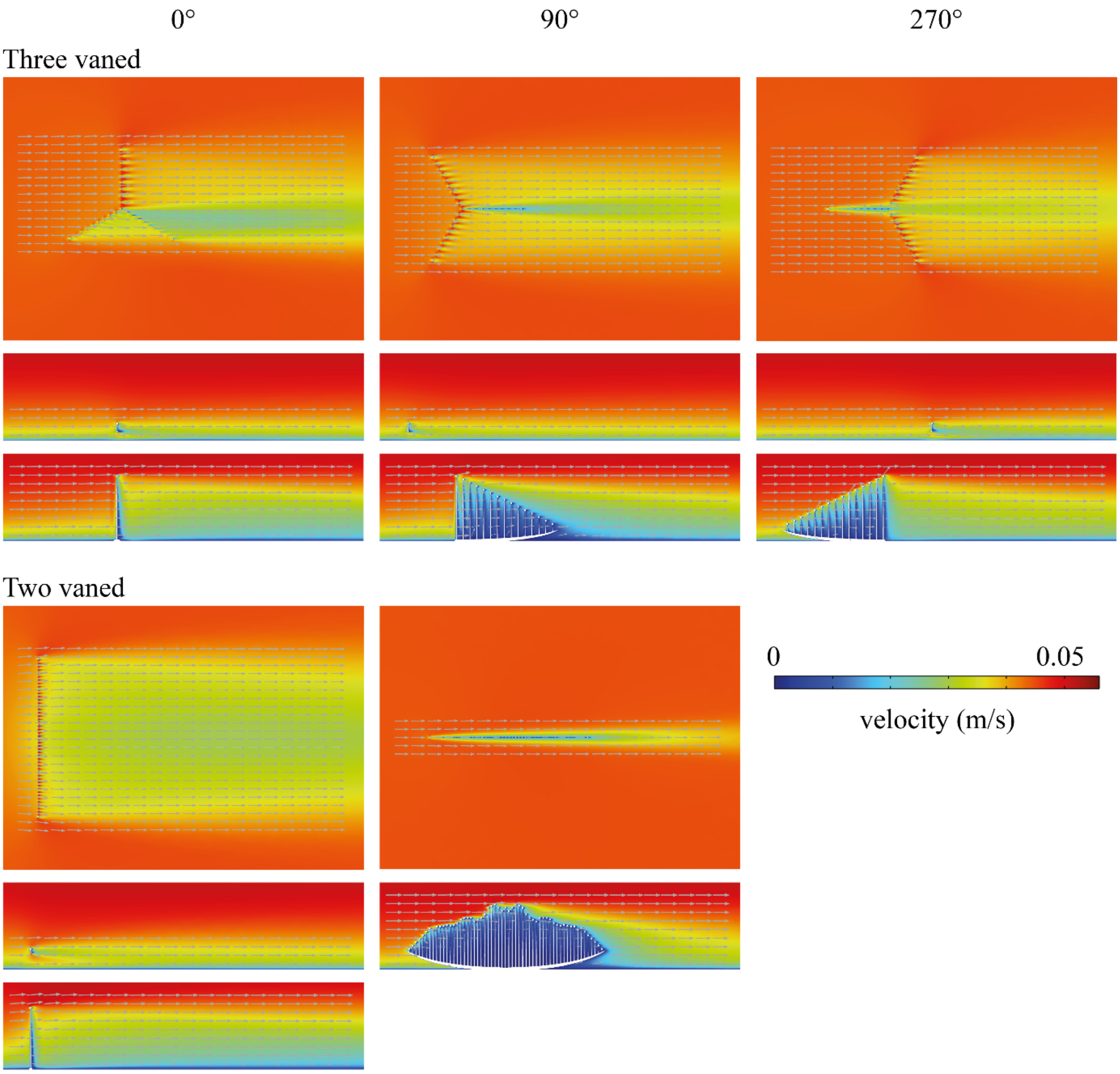

Figure S14. Two-dimensional plots (horizontal and vertical cross-sections) of velocity magnitude (*U*) with flow vectors (size of grey arrows proportional to natural logarithm of velocity magnitude) at an inlet velocity of 0.05 m/s for two *C. lyra* models, related to Figure 7. Direction of ambient flow from left to right.

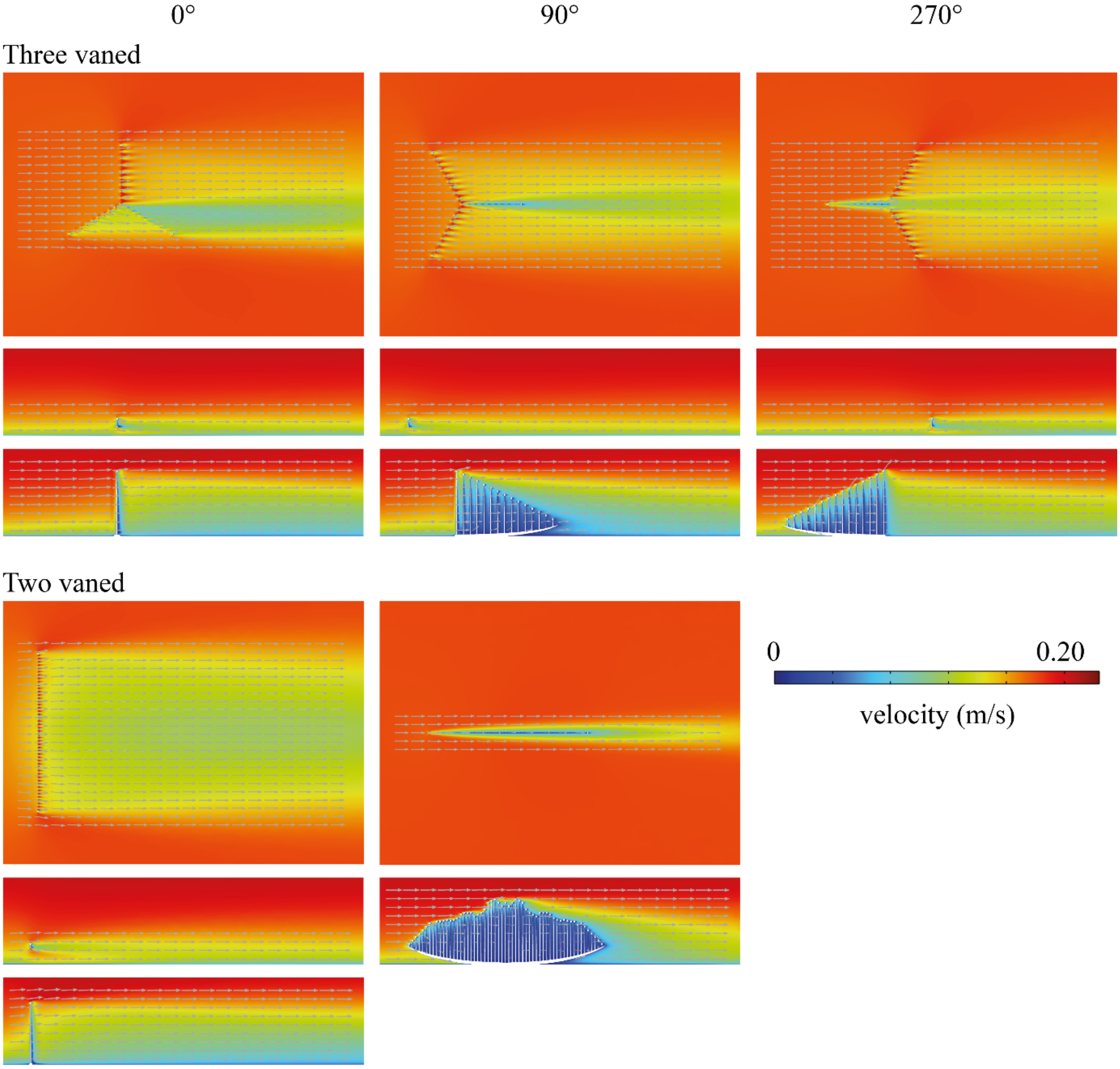

Figure S15. Two-dimensional plots (horizontal and vertical cross-sections) of velocity magnitude (*U*) with flow vectors (size of grey arrows proportional to natural logarithm of velocity magnitude) at an inlet velocity of 0.2 m/s for two *C. lyra* models, related to Figure 7. Direction of ambient flow from left to right.

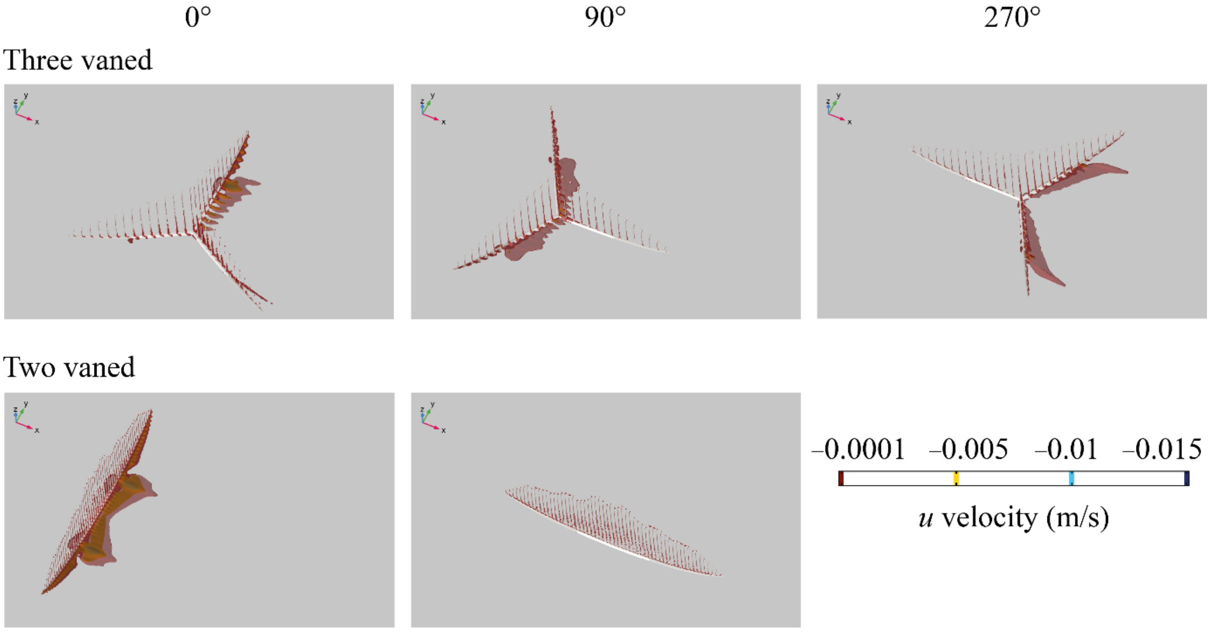

Figure S16. Three-dimensional isosurface plots of negative values of velocity component *u* (streamwise velocity) at an inlet velocity of 0.05 m/s for two *C. lyra* models, related to Figure 8. Direction of ambient flow from top left to bottom right.

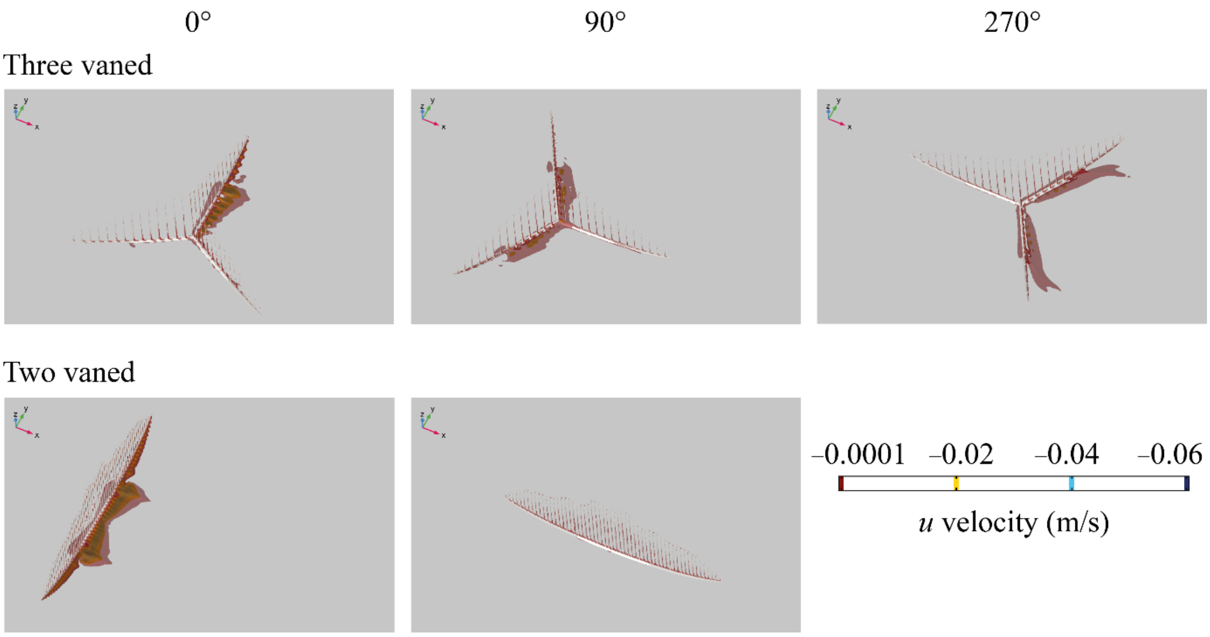

Figure S17. Three-dimensional isosurface plots of negative values of velocity component *u* (streamwise velocity) at an inlet velocity of 0.2 m/s for two *C. lyra* models, related to Figure 8. Direction of ambient flow from top left to bottom right.

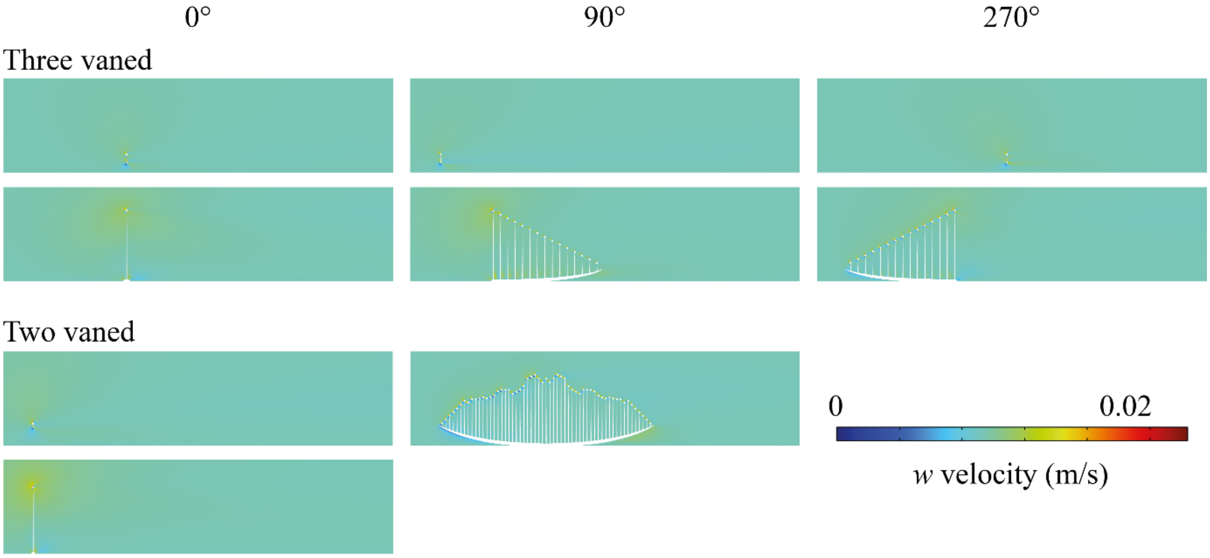

Figure S18. Two-dimensional plots (vertical cross-sections) of velocity component *w* (vertical velocity) at an inlet velocity of 0.05 m/s for two *C. lyra* models, related to Figure 7. Direction of ambient flow from left to right.

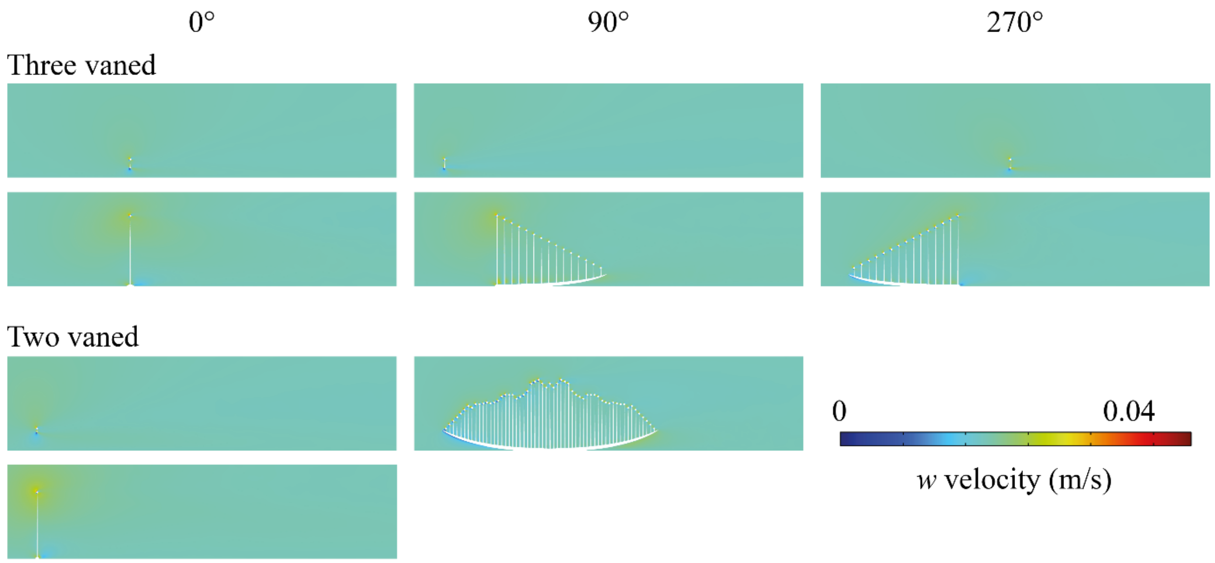

Figure S19. Two-dimensional plots (vertical cross-sections) of velocity component *w* (vertical velocity) at an inlet velocity of 0.1 m/s for two *C. lyra* models, related to Figure 7. Direction of ambient flow from left to right.

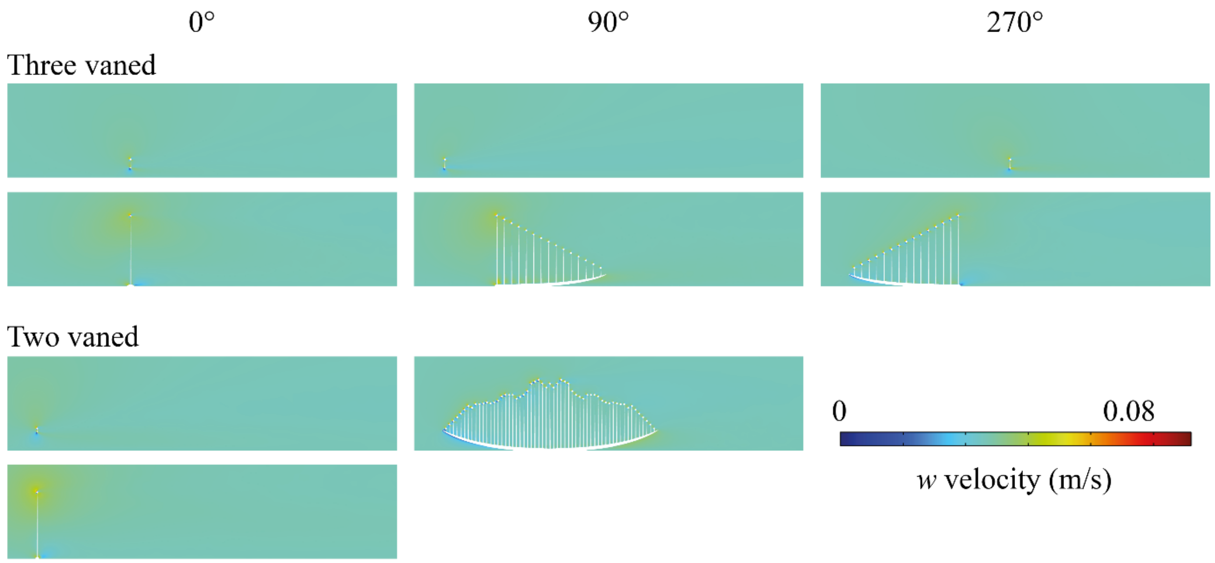

Figure S20. Two-dimensional plots (vertical cross-sections) of velocity component *w* (vertical velocity) at an inlet velocity of 0.2 m/s for two *C. lyra* models, related to Figure 7. Direction of ambient flow from left to right.
